# Glucocorticoids Suppress Mitochondrial Oxidant Production via Upregulation of Uncoupling Protein 2 in Hyperglycemic Endothelial Cells

**DOI:** 10.1371/journal.pone.0154813

**Published:** 2016-04-29

**Authors:** Domokos Gerö, Csaba Szabo

**Affiliations:** 1 Department of Anesthesiology, University of Texas Medical Branch, Galveston, Texas, United States of America; 2 University of Exeter Medical School, Exeter, United Kingdom; University of Ulm, GERMANY

## Abstract

Diabetic complications are the leading cause of morbidity and mortality in diabetic patients. Elevated blood glucose contributes to the development of endothelial and vascular dysfunction, and, consequently, to diabetic micro- and macrovascular complications, because it increases the mitochondrial proton gradient and mitochondrial oxidant production. Therapeutic approaches designed to counteract glucose-induced mitochondrial reactive oxygen species (ROS) production in the vasculature are expected to show efficacy against all diabetic complications, but direct pharmacological targeting (scavenging) of mitochondrial oxidants remains challenging due to the high reactivity of some of these oxidant species. In a recent study, we have conducted a medium-throughput cell-based screening of a focused library of well-annotated pharmacologically active compounds and identified glucocorticoids as inhibitors of mitochondrial superoxide production in microvascular endothelial cells exposed to elevated extracellular glucose. The goal of the current study was to investigate the mechanism of glucocorticoids' action. Our findings show that glucocorticoids induce the expression of the mitochondrial UCP2 protein and decrease the mitochondrial potential. UCP2 silencing prevents the protective effect of the glucocorticoids on ROS production. UCP2 induction also increases the oxygen consumption and the “proton leak” in microvascular endothelial cells. Furthermore, glutamine supplementation augments the effect of glucocorticoids via further enhancing the expression of UCP2 at the translational level. We conclude that UCP2 induction represents a novel experimental therapeutic intervention in diabetic vascular complications. While direct repurposing of glucocorticoids may not be possible for the therapy of diabetic complications due to their significant side effects that develop during chronic administration, the UCP2 pathway may be therapeutically targetable by other, glucocorticoid-independent pharmacological means.

## Introduction

Endothelial dysfunction has been implicated in the development of diabetic macrovascular and microvascular diseases **[1]**. Long-term clinical trials confirmed that improved glycemic control reduces the cardiovascular mortality and the risk of cardiovascular events including nonfatal myocardial infarction and stroke both in type I and type II diabetes **[2, 3]** suggesting that glucose itself is responsible for the vascular complications. As a downstream effector of hyperglycemia oxidative stress is involved in the development of vascular dysfunction. Glucose can induce reactive oxygen species (ROS) generation by multiple mechanisms: by activating the cytoplasmic NADPH oxidase, the polyol and hexosamine pathways, xanthine oxidase, or via the mitochondrial respiratory chain and even by inducing glucose auto-oxidation **[4]**. The endogenous antioxidant system should counterbalance the ROS production in the vasculature, but it fails to do so in diabetes, leading to oxidative stress, which is considered a key step in the pathogenesis of endothelial dysfunction **[4–9]**. While good glycemic control could prevent ROS generation, it is hard to achieve it continuously in all patients due to stress conditions (e.g. infections) or non-compliance. Supplementation of natural antioxidants and free radical scavengers have been tested to reduce the oxidative damage, but they provide limited protection due to their very short half-life **[10–15].** Inhibitors of the enzymatic sources of ROS generation **[16–22]** or the downstream effectors **[23, 24]** also showed modest beneficial effect in preclinical studies confirming the involvement of these oxidative stress pathways. By comparing the efficacy of various experimental therapeutic approaches against diabetic complications, Calcutt et al. found that those interventions that target the vasculature show beneficial effects against all complications **[25].** Therapies that target the macrovessels (eg. antihypertensive drugs) show limited protection against some complications, while those that target oxidative stress and effect both the macro- and the microvasculature are similarly effective against all complications. Thus, to develop novel therapies that can supplement current interventions, microvascular oxidative damage may be targeted.

Recently, mitochondrial superoxide production has been proposed to serve as an upstream component that activates both the polyol pathway and the advanced glycation endproduct (AGE) formation leading to inflammation and tissue damage **[4, 6, 26].** The significance of mitochondrial ROS production and dysfunction is further supported by its essential role in the cellular ATP generation and by its contribution to the cellular energy metabolism. Elevated glucose level may increase the mitochondrial proton gradient and result in higher superoxide ‘leakage’ from the respiratory chain in endothelial cells **[27].** To test whether the mitochondrial ROS generation can be directly addressed, we conducted a cell-based screening campaign and tested >6,000 compounds to reduce the glucose-induced mitochondrial superoxide production in endothelial cells **[28].** Our chemical genomics approach identified the selective serotonin reuptake inhibitor (SSRI) paroxetine, the microtubular agents colchicine and nocodazole and the group of glucocorticoid steroids as inhibitors of the mitochondrial ROS production. We conducted further experiments to uncover the mechanism of action of glucocorticoid steroids, and here we report that steroids induce UCP2 expression in microvascular endothelial cells and reduce the mitochondrial ROS generation by normalizing the mitochondrial membrane potential. This newly identified mechanism of action of glucocorticoid steroids can serve as the basis for drug discovery approaches to target the glucose-induced endothelial dysfunction.

## Methods

### Cell culture

b.End3 murine microvascular endothelial cells were obtained from the European Collection of Cell Cultures (ECACC, Salisbury, UK). The b.End3 cells were established form brain endothelial cells of 129/Sv mice by immortalization with the Polyoma virus middle T-antigen **[29]**. The cells were maintained in Dulbecco’s modified Eagle’s medium (DMEM) (Biochrom AG, Berlin, Germany) containing 1g/l glucose supplemented with 10% fetal bovine serum (FBS, Hyclone, Logan, UT), 1% non-essential amino acids, 100 IU/ml penicillin and 100 μg/ml streptomycin (Invitrogen, Carlsbad, CA) at 37°C in 10% CO_2_ atmosphere **[28]**. bEnd.3 microvascular cells were purchased from the American Type Culture Collection (ATCC, Manassas, VA) and maintained similarly. This cell line was established by infecting BALB/c cortical capillary endothelial cells with the Polyoma middle T-antigen expressing retrovirus **[30, 31]**. EA.hy926 human endothelial cells were obtained from ATCC (Manassas, VA) and maintained in DMEM containing 1 g/l glucose supplemented with 10% FBS, 1% non-essential amino acids, 100 IU/ml penicillin and 100 μg/ml streptomycin. HepG2 human hepatocellular carcinoma cells were obtained from ATCC (Manassas, VA) and maintained in Eagle’s minimum essential medium (EMEM, Cellgro Mediatech Inc., Manassas, VA) supplemented with 10% FBS, 100 IU/ml penicillin and 100 μg/ml streptomycin.

### Hyperglycemia-induced mitochondrial ROS production and compound treatments

Mitochondrial ROS generation can be induced by prolonged exposure to high glucose in endothelial cells as we previously described **[28]**. Microvascular endothelial cells (20 000/well) were plated into 96-well tissue culture plates and were cultured for 24 hours. Hyperglycemia (40mM glucose) was initiated by replacing the culture medium with fresh DMEM containing 7.2 g/l glucose supplemented with 10% FBS, 1% non-essential amino acids, 100 IU/ml penicillin and 100 μg/ml streptomycin and the cells were exposed to high glucose level for 10 days if not noted otherwise. The culture medium was supplemented with pyruvate (10 mM) as fresh source of energy after 4 days of exposure.

EA.hy926 cells were used in a similar manner but were exposed to hyperglycemia in medium 199 (M199, Sigma-Aldrich, St. Louis, MO) supplemented with 15% FBS, 4 mM glutamine, 7.5 U/ml heparin, 2.5 μg/ml human endothelial cell growth factor (ECGF, R&D Systems, Minneapolis, MN), 2 ng/ml human epidermal growth factor (EGF, R&D Systems, Minneapolis, MN), 100 IU/ml penicillin and 100 μg/ml streptomycin.

Dexamethasone and mifepristone were purchased from Sigma-Aldrich (St. Louis, MO) and were dissolved at 10 mM in dimethyl sulfoxide (DMSO). Dilutions were made in DMSO and phosphate buffered saline (PBS) to administer the compounds in 1/20 culture volume with final DMSO concentration of 0.5%. The cells were treated with the compounds for 3 days by administering the drugs on the 7^th^ days of the hyperglycemic exposure unless otherwise stated.

### ROS production and viability

Μeasurements of the mitochondrial superoxide generation by MitoSOX Red and the cellular reactive oxygen species (ROS) production by CM-H_2_DCFDA were previously described **[28]**. After the hyperglycemia exposure the cells were loaded with the mitochondrial superoxide sensor MitoSOX™ Red (2.5 μM, Life Technologies, Carlsbad, CA) or with the cell-permeable ROS indicator 5-(and-6)-chloromethyl-2',7'-dichlorodihydrofluorescein diacetate (CM-H_2_DCFDA, 10 μM, Life Technologies, Carlsbad, CA) and DNA stain Hoechst 33342 (10 μM) for 25min. Reading medium (PBS supplemented with 1 g/l glucose and 10% bovine growth serum (BGS, Hyclone, Logan, UT) was added to the cells and the oxidation of MitoSOX™ Red (Ex/Em: 530/590nm) or CM-H_2_DCFDA (Ex/Em: 485/528 nm) was recorded kinetically on Synergy 2 plate reader (BioTek, Winooski, VT) at 37°C for 35 min. ROS production is shown as the Vmax value of the fluorescence probe oxidation or as percent values of Vmax values of control cells. The fluorescence of Hoechst 33342 (Ex/Em: 360/460 nm) was used to calculate the viability of the cells using a calibration curve created by serial dilution of the cells.

### Mitochondrial membrane potential

The mitochondrial potential was measured with JC-1 (Sigma-Aldrich, St. Louis, MO) fluorescent probe as previously described **[32]**. The cells were loaded with the dye by exposing them to JC-1 stain solution containing 10 μM JC-1 and 0.6 mM β-cyclodextrin (Sigma-Aldrich, St. Louis, MO) in OptiMEM I medium at 37°C for 30 min. Subsequently, the cells were washed in phosphate buffered saline (PBS) and the red (Ex/Em: 485/528nm) and green (Ex/Em: 530/590nm) fluorescence was measured on a microplate reader (Synergy 2, Biotek, Winooski, VT, USA). The mitochondrial potential is reported as percent values of the ratio of the mitochondrial J-aggregates (red fluorescence) and the cytoplasmic monomer form of the dyes (green fluorescence) compared to vehicle-treated normoglycemic control cells.

Changes in the mitochondrial potential were also investigated by measuring the uptake of Mitotracker Green FM (Life Technologies, Carlsbad, CA). The uptake of the Mitotracker Green FM is potential-sensitive but it is less sensitive for rapid changes than JC-1 **[33]**. The cells were loaded with Mitotracker Green FM (0.5 μM) and Hoechst 33342 (10 μM) in PBS at 37°C for 30 min, then the cells were washed twice and the fluorescence of Mitotracker Green (Ex/Em: 485/528 nm) and Hoechst 33342 (Ex/Em: 360/460 nm) was recorded on Synergy 2 reader (BioTek, Winooski, VT).

### MTT and LDH assays, ATP measurement

The MTT assay and LDH activity measurements were performed as previously described **[34, 35].** Briefly, the cells were incubated in medium containing 0.5 mg/mL 3-(4,5-dimethyl-2-thiazolyl)-2,5-diphenyl-2H-tetrazolium bromide (MTT, Calbiochem, EMD BioSciences, San Diego, CA) for 1 hour at 37°C at 10% CO_2_ atmosphere. The converted formazan dye was dissolved in isopropanol and the absorbance was measured at 570 nm. Serial dilution of the cells was used to fit a curve on the absorbance values. MTT conversion rate values are shown as percent values relative to normoglycemic controls.

Total LDH content of the cells was measured by lysing the cells in 0.15 M saline containing 1% Triton-X-100 (30 μl/well) and measuring the LDH activity by adding 100 μl LDH assay reagent containing 110 mM lactic acid, 1350 mM nicotinamide adenine dinucleotide (NAD^+^), 290 mM *N*-methylphenazonium methyl sulfate (PMS), 685 mM 2-(4-Iodophenyl)-3-(4-nitrophenyl)-5-phenyl-2*H*-tetrazolium chloride (INT) and 200 mM Tris (pH 8.2). The changes in absorbance were read kinetically at 492 nm for 15 min (kinetic LDH assay). LDH activity values are shown as percent values relative to cells maintained under normoglycemia.

ATP concentration was determined by the commercially available CellTiter-Glo® Luminescent Cell Viability Assay (Promega, Madion, WI). The cells were lysed in 100 μL of CellTiter-Glo reagent according to the manufacturer’s recommendations and the luminescent signal was recorded for 1 s on a high sensitivity luminometer (Synergy Mx, Biotek, Winooski, VT, USA). The assay is based on ATP requiring luciferen-oxyluciferin conversion mediated by a thermostable luciferase that generates a stable “glow-type” luminescent signal. ATP standard (dilution series) was used to calculate the cellular ATP amount and the ATP values are shown as percent values of the normoglycemic controls.

### siRNA mediated gene silencing and real-time PCR measurements

Gene silencing and RNA level gene expression measurements were performed as previously described **[35]**. b.End3 cells (20,000/well) were plated on 96-well plates, the following day the cells were transfected with uncoupling protein 2 (UCP2) siRNA (1 pmol/well, Silencer Select, assay ID: s75721, Life Technologies, Carlsbad, CA) using Lipofectamine 2000 transfection reagent. Control cells were transfected with Silencer Select negative control #1 siRNA (ID: 4390844, Life Technologies, Carlsbad, CA). The knockdown efficiency was evaluated by real time PCR to confirm that the silencing lasts for 10 days.

Total RNA was isolated using a commercial RNA purification kit (SV total RNA isolation kit, Promega, Madison, WI). 2 μg RNA was reverse transcribed using the High Capacity cDNA Archive kit (Applied Biosystems, Foster City, CA) as previously described **[28, 35, 36]**. UCP2 expression was measured using species-specific UCP2 Taqman assays (murine assay ID: Mm00627598_m1, human assay ID: Hs01075227_m1, Life Technologies, Carlsbad, CA) and VIC-labeled 18S rRNA control reagents (Cat# 4308329 for murine and Cat# 4310893E for human samples, Life Technologies, Carlsbad, CA) for normalization on a CFX96 thermocycler (Bio-Rad, Hercules, CA). The expression of uncoupling protein 3 (UCP3) was measured by the following Taqman assay: assay ID: Mm01163394_m1 (Life Technologies, Carlsbad, CA).

### Mitochondria isolation, western blotting

Endothelial cells were exposed to hyperglycemia or maintained under normoglycemic conditions for 10 days and the mitochondria were isolated using the Mouse Mitochondria Isolation Kit (Miltenyi Biotec Inc., Auburn, CA). The cells were washed in PBS, scraped in separation buffer and lysed using a Dounce homogenizer. The lysates were incubated with anti-TOM22 microbeads (1:200) at 4°C for 1 hour to magnetically label the mitochondria. Then the labeled mitochondria were captured on a MACS separation column and washed with separation buffer. Finally, mitochondria were eluted by flushing the column with 1.5 ml buffer, pelleted and resuspended in 100 μl storage buffer. Mitochondria samples (10 μg protein) were heated to 37°C for 5 min. and resolved on 4–12% NuPage Bis-Tris acrylamide gels (Invitrogen, Carlsbad, CA) then transferred to nitrocellulose. Membranes were blocked in 10% non-fat dried milk and probed overnight with MitoProfile Total OXPHOS Rodent WB Antibody Cocktail (1:500, MitoSciences/Abcam, Cambridge, MA). The Antibody Cocktail contains antibodies against the following proteins in the respective respiratory complexes: NADH dehydrogenase (Ubiquinone) 1 beta subcomplex, 8 (NDUFB8, Complex I), Succinate dehydrogenase [ubiquinone] iron-sulfur subunit (SDHB, Complex II), Ubiquinol-Cytochrome c reductase Core Protein II (UQCRC2, Complex III), Cytochrome c oxidase subunit I (MTCO1, Complex IV), ATP synthase subunit alpha (ATP5A1, Complex V). The antibodies in the cocktail detect subunits that are labile when the complexes are not assembled.

Cell samples were lysed in denaturing loading buffer (20 mM Tris, 2% SDS, 10% glycerol, 6 M urea, 100 μg/ml bromophenol blue, 200 mM ß-mercaptoethanol) **[35, 36]**. Lysates were sonicated, boiled and resolved on 4–12% NuPage Bis-Tris acrylamide gels (Invitrogen, Carlsbad, CA), then transferred to nitrocellulose. Membranes were blocked in 10% non-fat dried milk and probed overnight with UCP-2 antibody (1:100, Santa Cruz Biotechnology Inc., Dallas, TX). After incubation with peroxidase conjugates (Cell Signaling, Danvers, MA) the blots were detected on a CCD-camera based detection system (GBox, Syngene USA, Frederick, MD) with enhanced chemiluminescent substrate. To normalize the signals, membranes were reprobed with horseradish peroxidase labeled actin antibody (1:3000, Santa Cruz Biotechnology Inc., Dallas, TX). The signals were quantitated using Genetools analysis software (Syngene USA, Frederick, MD).

### Extracellular Flux Analysis

An XF24 Analyzer (Seahorse Biosciences, Billerica, MA) was used to measure metabolic changes in b.End3 cells **[28, 37, 38]**. The XF24 creates a transient 7 μl chamber in specialized microplates that allows real-time measurement of oxygen and proton concentration changes via specific fluorescent dyes and calculates OCR (oxygen consumption rate) and PPR (proton production rate), measures of mitochondrial respiration and glycolytic activity. The proton production rate is expressed in pMol/min, while ECAR is in pH/min. The OCR and PPR values represent the metabolism of cells, but may also reflect the number of viable cells.

b.End3 cells were transfected with UCP2 or control siRNA and exposed to hyperglycemia for 5 days as described above. The culture medium was changed to unbuffered DMEM (pH 7.4) containing 5 mM glucose, 2 mM L-glutamine and 1 mM sodium pyruvate to allow measurement of the proton production. After determining the basal OCR and PPR values, the cells were treated with mifepristone (3 μM) and the OCR and PPR values were monitored for 8 hours. Subsequently oligomycin, FCCP and antimycin A were injected through the ports of the Seahorse Flux Pak cartridge to reach final concentrations of 1 μg/ml, 0.3 μM and 2 μg/ml, respectively, to determine the amount of oxygen consumption linked to ATP production, the level of non-ATP-linked oxygen consumption (proton leak) as well as the maximal respiration capacity and the non-mitochondrial oxygen consumption.

### Statistics

One-way analysis of variance (ANOVA) was used to detect differences between groups. Post hoc comparisons were made using Tukey's test. A value of *p* < 0.05 was considered statistically significant. All statistical calculations were performed using Prism 6 analysis software (GraphPad Software, Inc., La Jolla, CA). Data are shown as mean ± SEM values.

## Results

### High glucose exposure induce mitochondrial hyperpolarization in b.End3 microvascular endothelial cells

**Fig 1:**
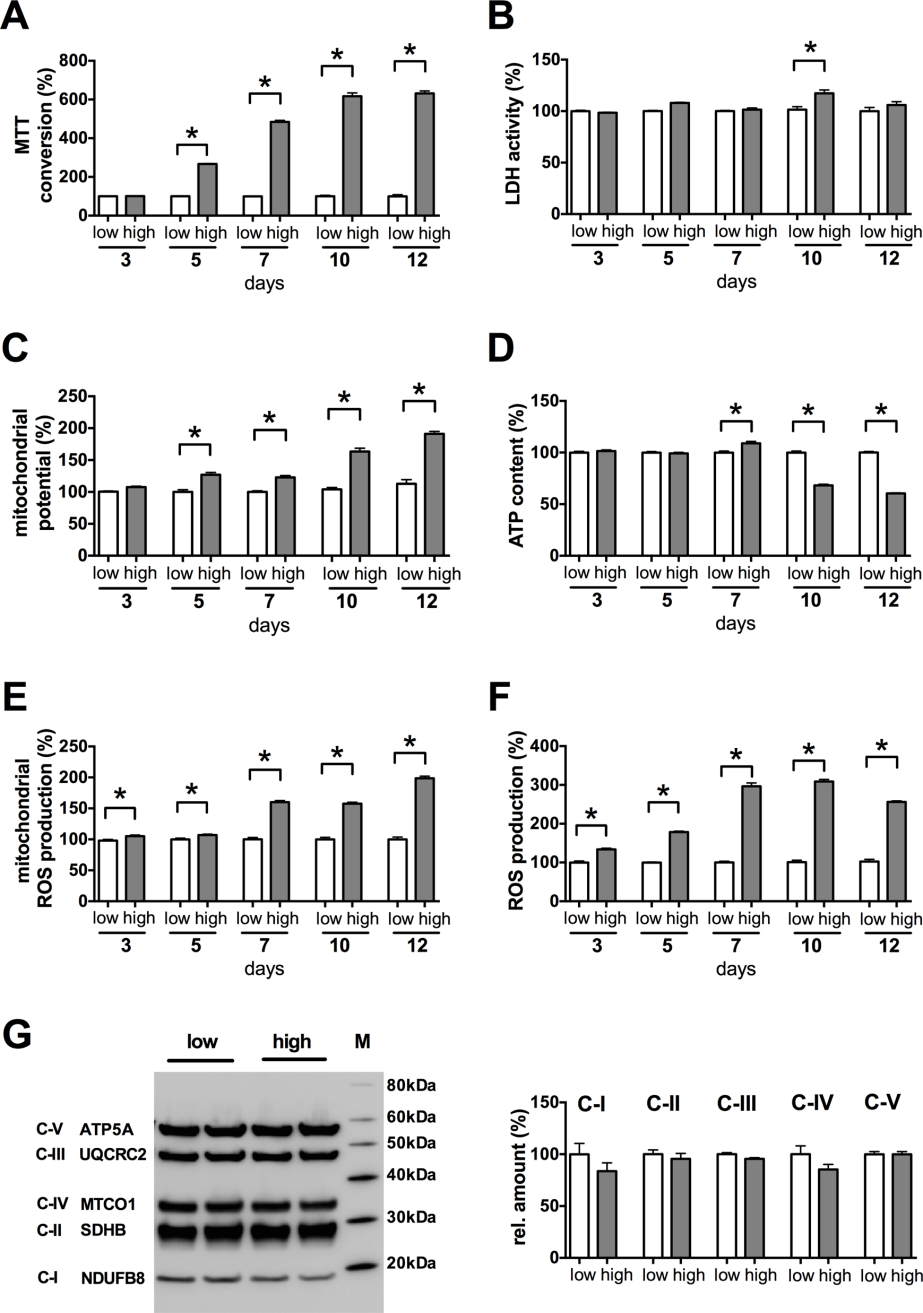
High glucose exposure induces mitochondrial hyperpolarization and oxidant production in b.End3 microvascular endothelial cells. **A-F:** Confluent b.End3 endothelial cells were maintained in low or high-glucose containing medium for 3–12 days and metabolic indices and ROS production were determined. **A:** The mitochondrial citric acid cycle activity was determined by measuring the MTT converting capacity of the cells. **B:** The anaerobic metabolic capacity of the cells was determined by measuring the LDH activity of the cells. **C:** The mitochondrial membrane potential was determined by JC-1 dye. **D:** The cellular ATP content was measured. **E, F:** The cellular ROS production was determined by (**E**) measuring the mitochondrial superoxide generation and (**F**) the cellular H_2_O_2_ production. **G:** Respective levels of the mitochondrial respiratory complexes were determined by MitoProfile total OXPHOS antibody cocktail. Representative blot image and densitometric analysis results are shown. (*p<0.05 high-glucose exposure induced significant changes compared to cells maintained in low glucose containing medium.)

To uncover the mechanism of action of glucocorticoid steroids against the glucose-induced mitochondrial ROS generation we characterized the major metabolic changes associated with the increased ROS production in the b.End3 cells exposed to hyperglycemia. b.End3 microvascular endothelial cells show a progressive increase in the mitochondrial MTT conversion but there is no change in the lactate dehydrogenase (LDH) activity when exposed to hyperglycemia (**Fig 1A and 1B**). MTT is believed to be converted predominantly by the mitochondrial succinate dehydrogenase enzyme (respiratory complex II) **[39]**, thus the MTT assay primarily depicts the oxidative phosphorylation (OXPHOS) and the aerobic metabolism of the cells, whereas LDH is a key enzyme in the anaerobic metabolism. The mitochondrial membrane potential shows a similar progressive increase in the cells as the MTT conversion (**Fig 1C**) with significantly increased values after 5 days of exposure. These changes have no effect on the cellular ATP content. The cells maintain a stable energy level until the 10^th^ day of exposure, when they start to show a decline in the ATP level (**Fig 1D**). While the cellular LDH content (anaerobic metabolism) increases at that time (**Fig 1B**), it is not sufficient to reinstate the cellular energy level (**Fig 1D**).

Both the mitochondrial and the cytoplasmic ROS production increases in the cells exposed to hyperglycemia, as measured by the mitochondrial superoxide-sensitive MitoSOX Red probe and the H_2_O_2_-sensitive 5-(and-6)-chloromethyl-2',7'-dichlorodihydro-fluorescein diacetate, acetyl ester (CM-H_2_DCFDA) (**Fig 1E and 1F**). Significantly increased ROS production is detectable already on the 3^rd^ day of the hyperglycemic exposure, and on the subsequent days a progressive increase can be observed similar to the increase in the mitochondrial membrane potential. We previously reported that there is not much change in the expression of the mitochondrial metabolism associated genes in microvascular endothelial cells in response to high glucose exposure **[28]**. We tested, whether there is any change in the assembly of the mitochondrial respiratory complexes (**Fig 1G**), but we found no alteration in them. While there is no detectable change in the respiratory complex assembly, there seems to be a functional deficit in the mitochondrial electron transfer or in the chemiosmotic coupling, since the mitochondria fail to use the elevated membrane potential to produce more ATP or even to maintain the basal cellular energy level after prolonged exposure to high glucose. Thus, the increased glucose load leads to mitochondrial hyperpolarization that might be responsible for the increased ROS generation in the b.End3 cells.

### Glucocorticoids inhibit the mitochondrial ROS production in microvascular endothelial cells

**Fig 2:**
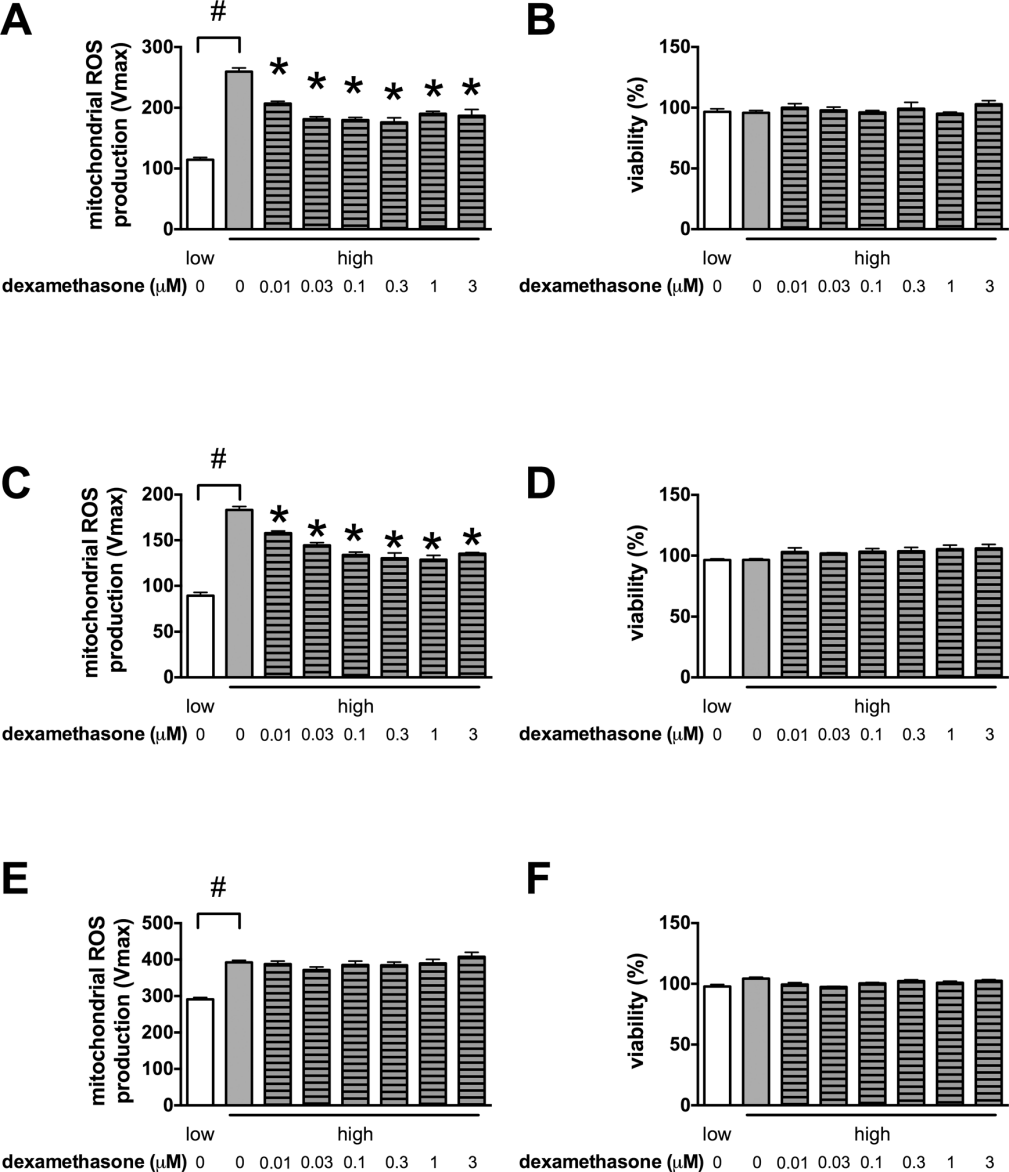
Glucocorticoids decrease the glucose-induced mitochondrial ROS production in microvascular endothelial cells. **A, B:** b.End3 microvascular endothelial cells were exposed to high glucose for 7 days and were treated with dexamethasone at the indicated concentrations for 3 days. **A:** ROS production was measured with the mitochondrial superoxide specific MitoSOX Red and **(B)** the viability was determined by measuring the Hoechst 33342 DNA dye uptake. **C, D:** b.End3 cells were exposed to high glucose for 7 days and were treated with mifepristone at the indicated concentrations for 3 days. **C:** ROS production was measured with the mitochondrial superoxide specific MitoSOX Red and **(D)** viability was determined by measuring the Hoechst 33342 DNA dye uptake. **E, F**: EA.hy926 human venous endothelial cells were exposed to high glucose for 7 days and treated with dexamethasone at the indicated concentrations for 3 days. **E**: The mitochondrial superoxide production was determined by MitoSOX Red stain and **F**: the viability by Hoechst 33342 DNA dye uptake. (#p<0.05 high-glucose exposure induced significant changes compared to cells maintained in low glucose containing medium,*p<0.05 glucocorticoid treatment significantly reduced the ROS production.)

**Fig 3:**
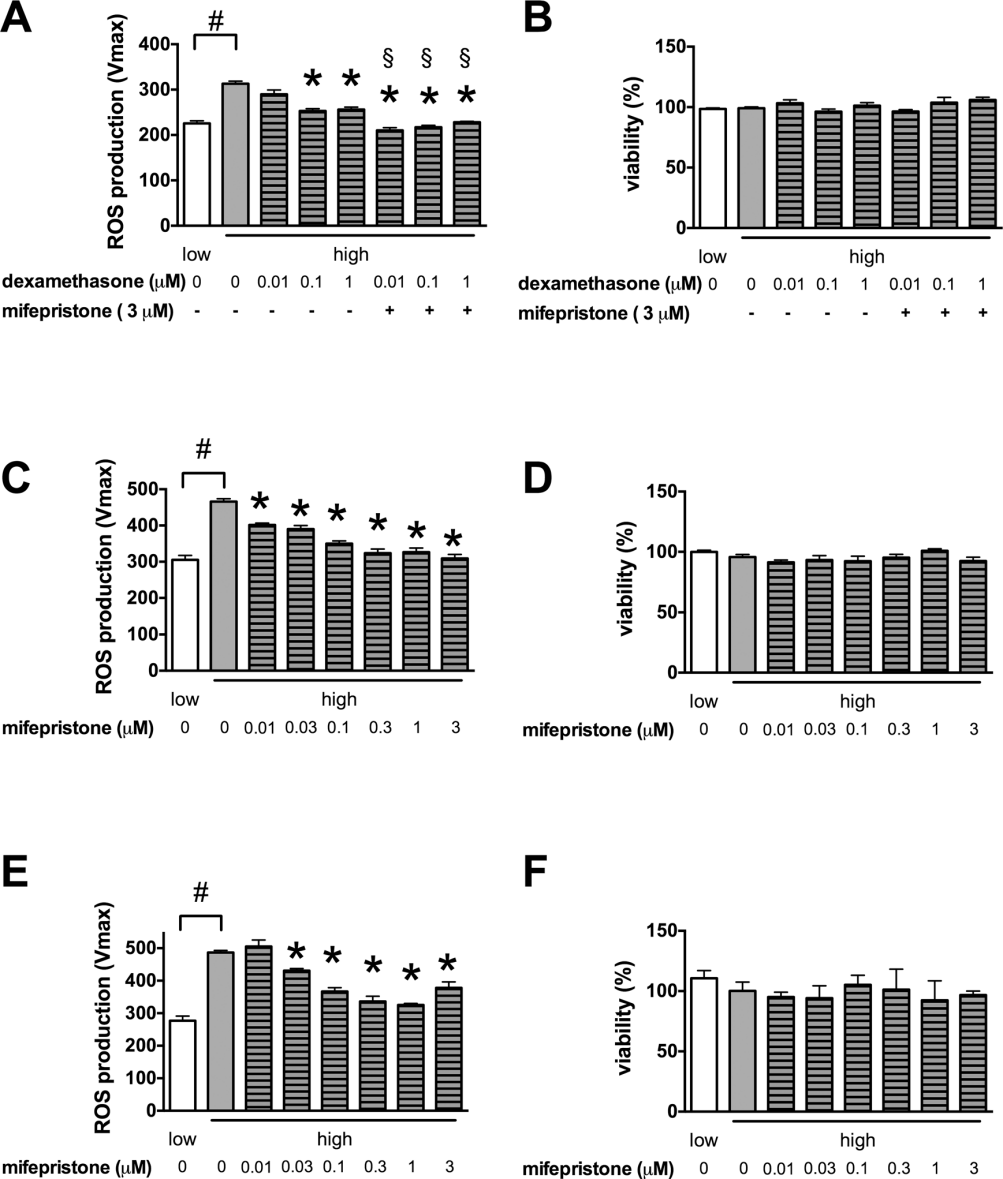
Mifepristone inhibits the glucose-induced mitochondrial ROS production in microvascular endothelial cells. **A-D:** b.End3 microvascular endothelial cells were exposed to high glucose for 7 days and were treated with dexamethasone and mifepristone (**A, B**) or with mifepristone alone (**C, D**) at the indicated concentrations for 3 days. **A, C:** The ROS production was measured with the mitochondrial superoxide specific MitoSOX Red **B, D:** The viability was determined by measuring the Hoechst 33342 DNA dye uptake. **E, F**: bEnd.3 BALB/c murine microvascular endothelial cells were exposed to high glucose for 7 days and were treated with mifepristone for 3 days at the indicate concentrations. **E**: The mitochondrial ROS production was measured by MitoSOX Red and **F**: the viability was determined by the Hoechst 33342 uptake. (#p<0.05 high-glucose exposure induced significant changes compared to cells maintained in low glucose containing medium,*p<0.05 glucocorticoid treatment significantly reduced the ROS production, §p<0.05 mifepristone induced significant reduction in the ROS generation compared to dexamethasone alone.)

We found that glucocorticoid steroids inhibit the glucose-induced mitochondrial ROS production in microvascular endothelial cells **[28]**. This novel anti-ROS action of glucocorticoids was clearly shared by the whole class of glucocorticoid steroid drugs tested. Flunisolide reduced the mitochondrial ROS production by 25%, budesonide by 23%, flurandrenolide by 19%, methylprednisolone by 17%, dexamethasone by 16% and betamethasone by 15% in our previous screen **[28]**. We found that dexamethasone reduced the high glucose-induced mitochondrial ROS production both in the Sv129-derived b.End3 and the BalbC-derived bEnd.3 microvascular endothelial cells in a concentration-dependent manner (**Fig 2A and 2C**) without affecting the cellular viability (**Fig 2B and 2D**). Dexamethasone reached its maximum effect in the nanomolar concentration range in microvascular endothelial cells, but it had no effect on the hyperglycemia-induced mitochondrial ROS production in the EA.hy926 venous endothelial cell line (**Fig 2E and 2F**). Next we tested the effect of the partial glucocorticoid receptor (GR) antagonist mifepristone on the ROS production inhibitory effect of dexamethasone in b.End3 cells. Unexpectedly, mifepristone did not block the effect of dexamethasone, but further reduced the mitochondrial ROS production in endothelial cells (**Fig 3A and 3B**). We found that mifepristone by itself decreased the hyperglycemia-induced ROS production in both microvascular endothelial cell lines (**Fig 3C–3F**), and at low micromolar concentrations it was more effective than dexamethasone. Interestingly, the antifungal ketoconazole and miconazole, that also show glucocorticoid receptor antagonist activity, similarly reduced the mitochondrial ROS production (by 9.6 and 18.7% respectively) in our previous screen **[28]**.

### Glucocorticoids restore the mitochondrial potential and induce UCP2 expression

**Fig 4:**
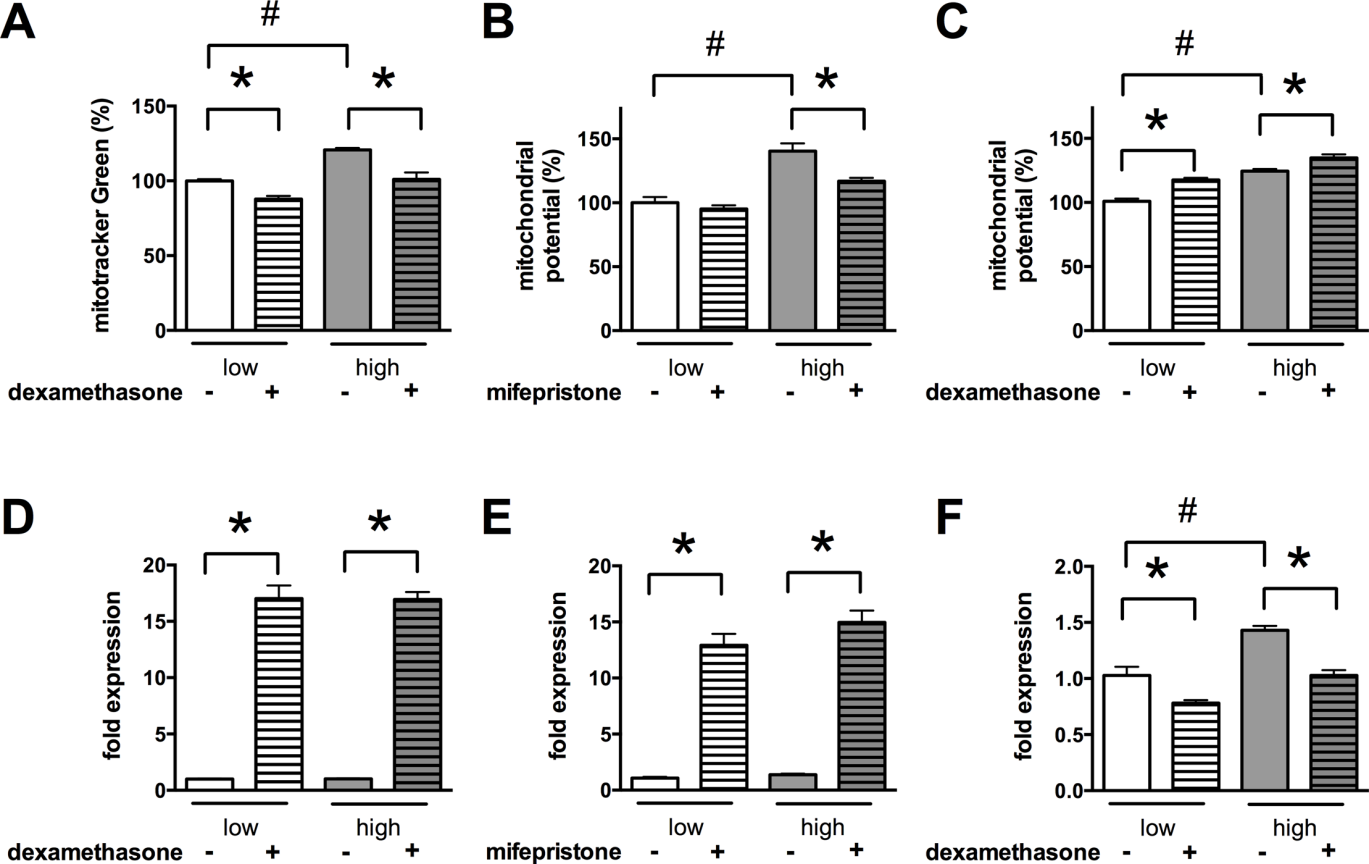
Glucocorticoid steroids block the glucose-induced mitochondrial hyperpolarization and induce UCP2 expression. **A, B, D, E:** b.End3 endothelial cells were exposed to high glucose for 7 days and treated with the dexamethasone (1 μM) or mifepristone (3 μM) for 3 days. **A, B:** Changes in the mitochondrial potential were determined by measuring the MitoTracker Green uptake (**A**) or the ratio of the mitochondrial J-aggregate and the free cytoplasmic form of JC-1 (**B**). **C, F**: EA.hy926 human venous cells were exposed to high glucose for 7 days and treated with dexamethasone (1 μM) for 3 days. **C**: The mitochondrial membrane potential was measured by JC-1 dye. **D, E, F**: UCP2 expression was determined by realtime PCR using Taqman assays. (#p<0.05 high-glucose exposure induced significant changes compared to cells maintained in low glucose containing medium,*p<0.05 glucocorticoid treatment induced significant changes.)

Since we found that hyperglycemia induces an increase in the mitochondrial potential, we tested whether the anti-ROS activity of glucocorticoids affects the mitochondrial potential. We found that both dexamethasone and mifepristone normalized the mitochondrial potential in b.End3 cells (**Fig 4A and 4B**), and no decrease but an increase was detectable in the EA.hy926 venous endothelial cells in which dexamethasone did not reduce the mitochondrial ROS production (**Fig 4C**). To test whether the uncoupling proteins are involved in the steroid-induced decrease in the mitochondrial potential we measured the expression of uncoupling protein 2 (UCP2) and 3 (UCP3). Both dexamethasone and mifepristone induced a ~10-fold increase in the expression of UCP2 at the mRNA level in b.End3 microvascular endothelial cells (**Fig 4D and 4E**). On the other hand, in the EA.hy926 venous endothelial cells, hyperglycemia by itself induced a ~1.5 fold increase in UCP2 expression and dexamethasone significantly reduced the expression of UCP2 both at the mRNA (**Fig 4F**) and at the protein level (**S1 Fig**). While glucocorticoids were found to induce the expression of UCP3 in muscle cells **[40]** we found that the expression of UCP3 was very low in the endothelial cells (threshold cycle values over 35 were measured) and remained unchanged in response to steroids suggesting a predominant role for UCP2 in the endothelial cells.

**Fig 5:**
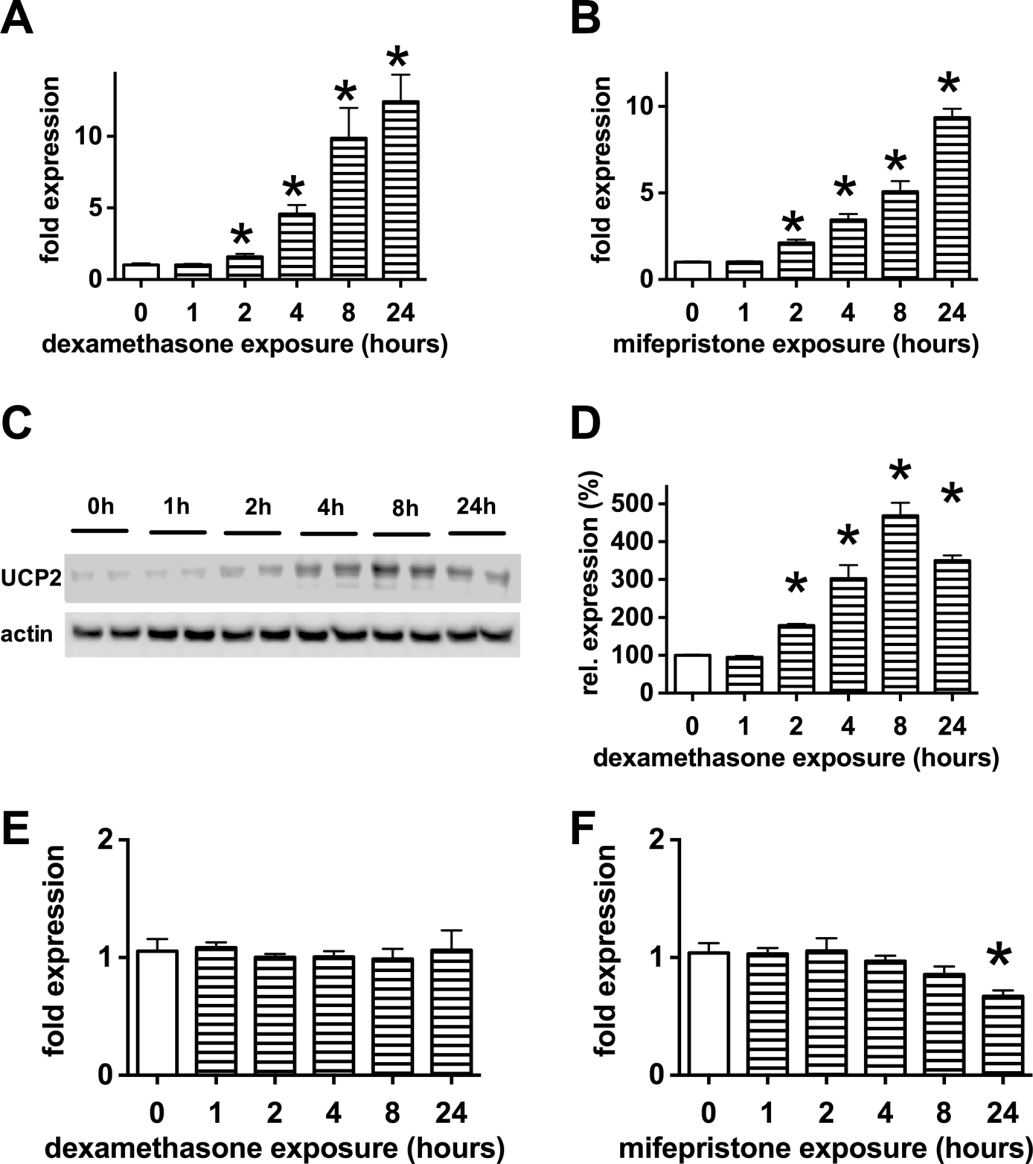
Time course of steroid induced UCP2 expression. **A-D:** Confluent b.End3 endothelial cells were exposed to **A, C, D**: dexamethasone (1 μM) or **B:** mifepristone (3 μM) for the indicated time period. **A, B**: UCP2 mRNA expression was measured by UCP2 Taqman assay using rRNA normalization. **C, D**: UCP2 protein expression was measured by Western blotting. Representative blot image (**C**) and densitometry results (**D**) are shown. **E, F:** HepG2 human liver cells were treated with dexamethasone (1 μM, **E**) or mifepristone (3 μM, **F**) for the indicated time periods and UCP2 expression was determined by Taqman assay. (*p<0.05 glucocorticoid treatment induced significant changes in the UCP2 expression.)

Next we investigated the time course of UCP2 induction by glucocorticoid steroids in b.End3 cells. We found that both dexamethasone and mifepristone induced UCP2 expression in a time-dependent fashion: the level of UPC2 mRNA doubled every two hours in the first 8 hours of steroid exposure with further increase measurable after 24 hours (**Fig 5A and 5B**). Similar changes were measured at the protein level in the first 8 hours, but no further increase was detectable at 24 hours (**Fig 5C and 5D**). Glucocorticoids steroids affected the UCP2 expression in microvascular endothelial cells at a concentration that is close to the circulatory levels of cortisol, so we tested whether they affect the expression of UCP2 in liver cells, the major site of energy metabolism. No change was detectable in UCP2 expression following dexamethasone treatment in HepG2 cells (**Fig 5D**), only a slight reduction was measurable in response to mifepristone (**Fig 5E**) suggesting that the glucocorticoid-induced UCP2 expression is not a universal phenomenon, but this effect is restricted to certain cell types including the microvascular endothelial cells.

**Fig 6:**
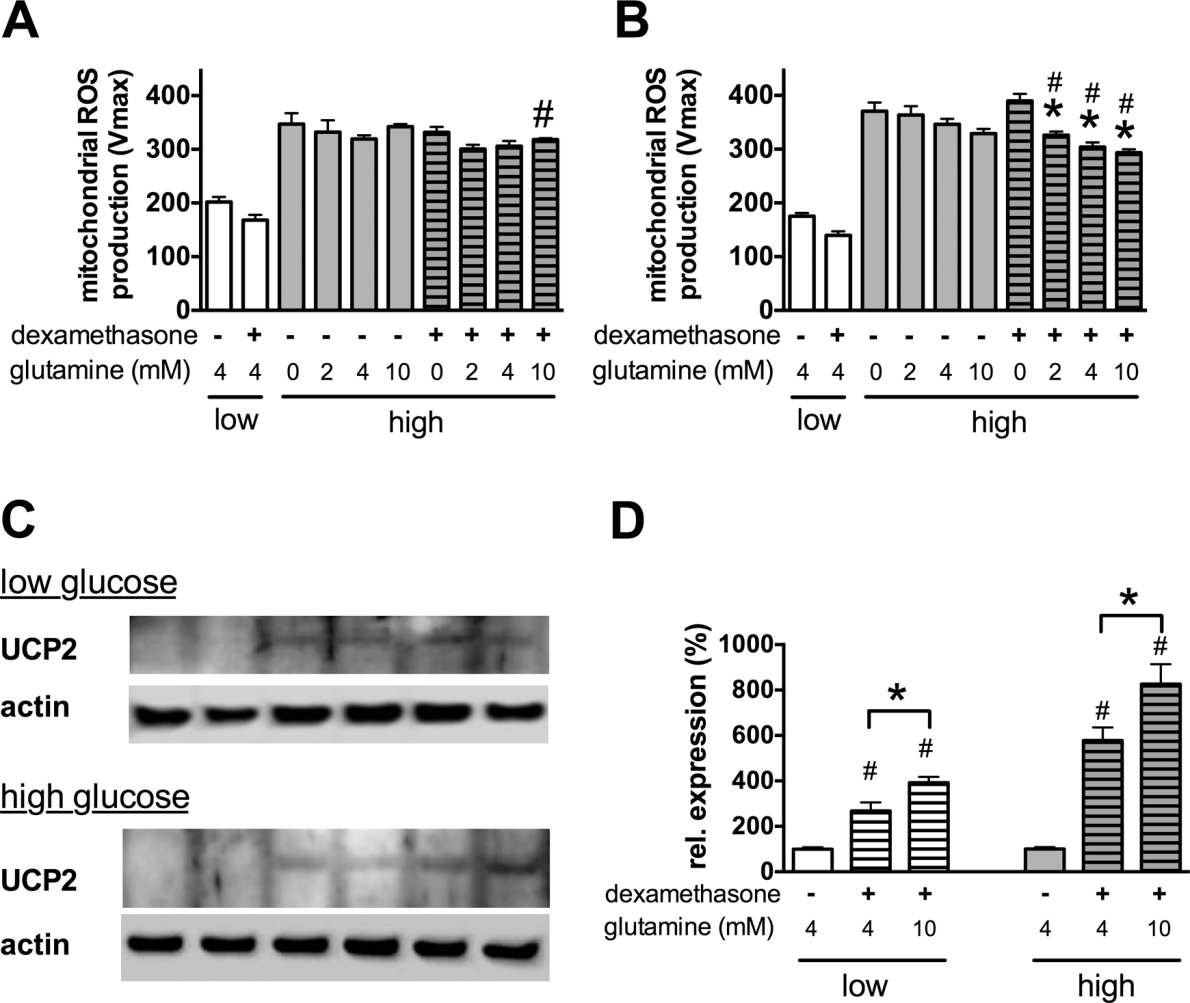
Glutamine potentiates the dexamethasone-mediated UCP2 induction and inhibition of ROS production. **A-D**: b.End3 cells were exposed to hyperglycemia for 7 days and were treated subsequently with dexamethasone (1 μM) and the indicated amount of glutamine for 6 hours (**A**) or 3 days (**B-D**). **C**: The mitochondrial superoxide production was measured by MitoSOX Red. **C-D**: UCP2 protein expression was measured by Western blotting. Representative blot image (**C**) and densitometric analysis results (**D**) are shown. (High-glucose exposure induced significant increase in the mitochondrial ROS production. #p<0.05 dexamethasone significantly decreased the ROS production compared to the high glucose group, *p<0.05 glutamine treatment resulted in a significant decrease in ROS production.)

Since UCP2 expression is regulated by glutamine availability at the translational level **[41]**, we tested whether the amount of glutamine affects the UCP2 expression and the mitochondrial ROS production in b.End3 cells. Restricting the glutamine amount or supplementing the culture medium with additional glutamine had little effect on the mitochondrial ROS production in the absence of glucocorticoids (**Fig 6A and 6B**), but it had a marked effect in combination with dexamethasone if the ROS production was measured after a longer treatment period. The complete removal of glutamine blocked the ROS inhibitory effect of dexamethasone and the extra glutamine potentiated the effect of dexamethasone (**Fig 6A and 6B**). As expected, the amount of UCP2 protein increased after the combined dexamethasone and glutamine treatment in the cells (**Fig 6C and 6D**).

Previous reports found that glucocorticoids may control the mitochondrial oxidative phosphorylation by multiple mechanisms: 1) by inducing the expression of nuclear-encoded OXPHOS genes, including cytochrome c **[42]** and cytochrome c oxidases 1–4 **[43–46]**, 2) by directly controlling the mitochondrial gene expression **[47]**, 3) by affecting the mitochondrial DNA replication **[48, 49]** and 4) by regulating the expression of UCP3 via a sirtuin 1 (SIRT)-mediated mechanism **[40]**. To test the possible contribution of the above actions of glucocorticoid steroids to the inhibitory effect on mitochondrial ROS generation, we measured the mitochondrial DNA (mtDNA) content of endothelial cells and the expression of previously identified target genes. We found that the hyperglycemic exposure reduced the mtDNA content of the cells and dexamethasone induced a significant reduction in the mtDNA content (**S2 Fig**). The expression of glucocorticoid receptor (GR) was suppressed in cells treated with dexamethasone confirming a negative feedback regulation (**S3A Fig**), but no reduction was measured in the expression of SIRT (**S3B Fig**), nor in the expression of the mitochondrial-encoded 16S RNA or COX3 genes (**S3C and S3E Fig**). While the complete depletion of the mtDNA may have an effect on the mitochondrial OXPHOS in the cells **[50, 51]**, we found that the expression of the mitochondrial-encoded genes (COX3, 16S RNA) remained unchanged, thus the reduction of the mtDNA content may not have a significant impact on the mitochondrial respiration and ROS production in b.End3 cells. The expression of the nuclear encoded electron carrier cytochrome c (Cyt C) was suppressed in dexamethasone-treated endothelial cells (**S3D Fig**). While the complete lack of cytochrome c causes deficiency in the respiration and leads to embryonic lethality in mice **[52, 53]**, the reduced expression may not be responsible for the reduced mitochondrial ROS production in the b.End3 cells, since we neither measured inhibition of the mitochondrial respiration nor a change in the cellular ATP content.

### UCP2 is responsible for the physiological mitochondrial membrane potential in microvascular endothelial cells and the anti-ROS action of glucocorticoids

**Fig 7:**
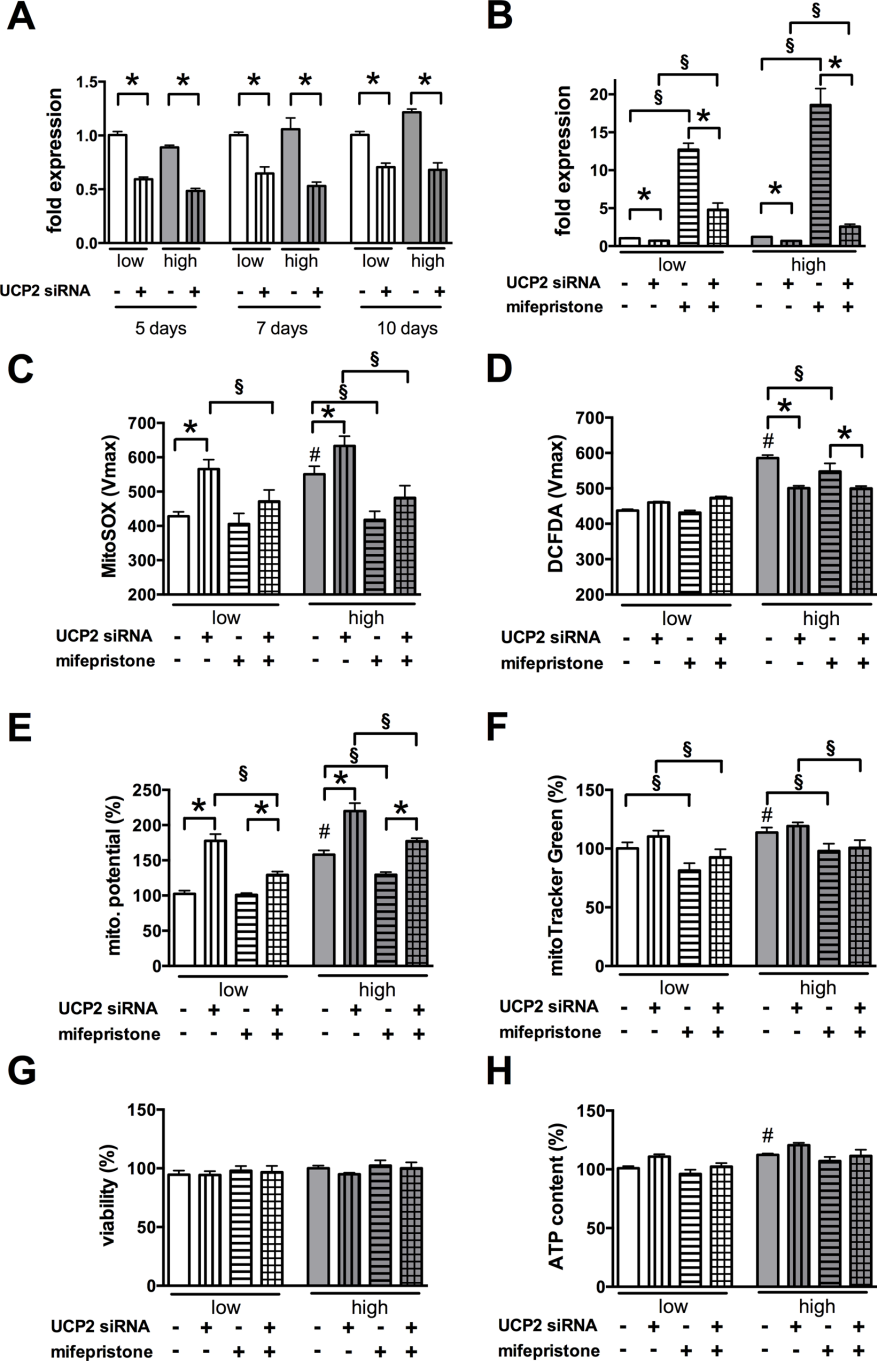
UCP2 silencing blocks the mifepristone-mediated UCP2 induction and antioxidant effects. **A-H:** b.End3 microvascular endothelial cells were transfected with UCP2 siRNA or negative control siRNA and exposed to high glucose for 5 days. Subsequently the cells were treated with mifepristone (3 μM) for 1 day. **A**: The mitochondrial superoxide production was measured by MitoSOX Red. **B**: The cellular H_2_O_2_ production was measured by CM-H_2_DCFDA. **C, D**: The mitochondrial membrane potential was measured by JC-1 (**C**) and also by the uptake of MitoTracker Green (**D**). **E**: The cellular viability was determined by measuring the Hoechst 33342 DNA dye uptake. **F**: The cellular ATP content was measured. **G**: The viability was determined by measuring the Hoechst 33342 DNA dye uptake. **H:** Cellular ATP content was determined. (#p<0.05 high-glucose exposure induced significant changes compared to cells maintained in low glucose containing medium,*p<0.05 UCP2 silencing induced significant changes in ROS production and in the mitochondrial potential, § p<0.05 mifepristone significantly reduced the ROS production and the mitochondrial potential.)

Since the above results suggested that the induction of UCP2 expression may be responsible for the glucocorticoid steroid-mediated anti-ROS effect in microvascular endothelial cells, we used siRNA-mediated gene silencing to examine the role of UCP2 in the mitochondrial potential and superoxide generation in hyperglycemic b.End3 cells. siRNA-mediated knockdown significantly reduced the expression of UCP2 both in normo- and hyperglycemic b.End3 cells for 10 days (**Fig 7A**) and partially blocked the steroid-induced UCP2 induction in the cells (**Fig 7B**). UCP2 silencing not only blocked the ROS-inhibitory effect of mifepristone but also caused an increase in the mitochondrial superoxide generation by itself (**Fig 7C**). Interestingly, it coincided with a decrease in the cytoplasmic ROS generation as measured with the H_2_O_2_-sensitive CM-H_2_DCFDA probe (**Fig 7D**). The reduced level of UCP2 caused an increase in the mitochondrial potential and partially blocked the membrane potential normalizing effect of mifepristone as measured by JC-1 (**Fig 7E**) and by MitoTracker Green FM uptake (**Fig 7F**). No change was detectable in the cell viability (**Fig 7G**) but the cellular ATP content showed an increasing tendency with the reduced UCP2 level (**Fig 7G**). Overall, these results suggest that UCP2 regulates the physiological mitochondrial membrane potential in microvascular endothelial cells and UCP2 expression is responsible for the decrease induced by glucocorticoid steroids in the hyperglycemic endothelial cells.

### UCP2 induction leads to an aerobic shift in the cellular metabolism in b.End3 microvascular endothelial cells

**Fig 8:**
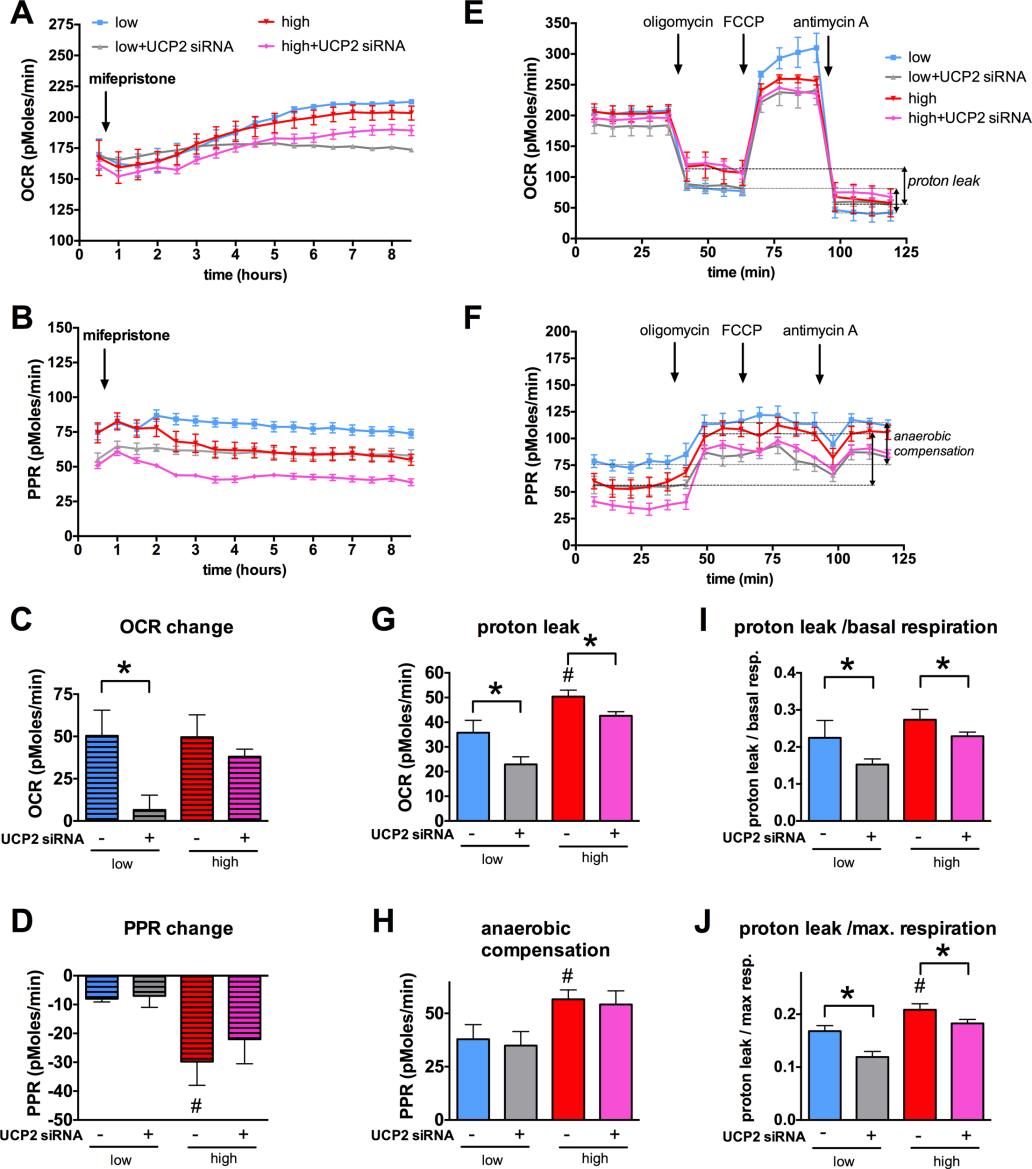
UCP2 silencing blocks the metabolic changes induced by mifepristone. **A-H:** b.End3 microvascular endothelial cells were transfected with UCP2 siRNA or negative control siRNA and exposed to high glucose for 5 days. **A-D**: The cells were treated with mifepristone (3 μM) and **A**: the cellular oxygen consumption rate (OCR) and **B**: proton production rate (PPR) was monitored in real time by the Seahorse XF24 Extracellular Flux Analysis system for 8 hours. **C, D**: The increase in the OCR values (**C**) induced by miferpristone and the decrease in PPR values (**D**) are shown. **E-H**: Subsequently, metabolic profiling of the cells was carried out by adding oligomycin, FCCP and antimycin A, respectively, with monitoring the changes in the OCR (**E**) and PPR (**F**) values. **G, H**: The non-ATP-linked oxygen consumption (proton leak) rate (**G**) and the anaerobic compensation (**H**) are shown. **I, J:** Proton link/basal respiration rate and proton link/maximal respiration rate values are shown. (#p<0.05 high-glucose exposure induced significant changes compared to cells maintained in low glucose containing medium,*p<0.05 UCP2 silencing significantly reduced the OCR increase and the proton leak.)

UCP2 was found to regulate the energy metabolism in stem cells **[54]** and the induction of UCP2 expression was shown to reduce the mitochondrial membrane potential and ROS production in A549 lung adenocarcinoma cells in which it also induced a shift in the cellular metabolism **[55]**. To test whether the glucocorticoid-mediated UCP2 induction also affects the metabolism in microvascular endothelial cells, we exposed the cells to hyperglycemia and followed the oxygen consumption and acid production in response to mifepristone. Mifepristone increased the oxygen consumption of the cells (**Fig 8A and 8C**) and slightly reduced the acid production (**Fig 8B and 8D**) resulting in an aerobic shift in the metabolism with a comparable time course to UCP2 induction. Following the steroid-mediated UCP2 induction, higher proton leak was measurable in the hyperglycemic cells (**Fig 8E, 8G and 8J**). UCP2 silencing partially blocked the increase of oxygen consumption and diminished the increase in the proton leak (**Fig 8E, 8G, 8I and 8J**). While no change was detectable in the basal oxygen consumption of the cells, the hyperglycemic cells showed higher increase in the anaerobic compensation following the inhibition of the mitochondrial respiration (**Fig 8F and 8H**). This effect may not be accounted to the increase in the proton leak, since in this respect the UCP2 silenced cells showed similar changes. Altogether, these results suggest that pharmacological induction of UCP2 expression cause distinct changes in the cellular metabolism in microvascular endothelial cells.

## Discussion

The key findings of the present study are the following:

1. Exposure to high extracellular glucose concentration induces mitochondrial hyperpolarization in endothelial cells.
2. Glucocorticoid steroids reduce the mitochondrial membrane potential and inhibit mitochondrial ROS generation in microvascular endothelial cells.
3. Glucocorticoids upregulate UCP2 in microvascular endothelial cells.
4. UCP2 functions as the main regulator of the mitochondrial potential in endothelial cells.

Glucose plays a fundamental part in the development of diabetic complications, still the mechanism, how this essential body fuel becomes an enemy, is controversial. The definition of “normoglycemia” has been often debated **[56, 57]** since the blood glucose level is not a constant value in healthy subjects. Based on the predictive value of glucose level for diabetic complications **[58]** blood sugar cut-off values were chosen that identify patients at risk for complications **[56, 59]**, and values below the cut-off values were accepted as normal. In diabetic patients the improved glycemic control was proven to prevent the development of vascular complications **[60–63]** confirming the pathogenic role of glucose. Thus, the maintenance of “normoglycemia” has become a widely accepted goal in diabetes management, but “glycemic control” by itself was not able to decrease the incidence of diabetic complications **[64]** suggesting that additional therapeutic modalities will be necessary. Oxidative stress as a well-recognized contributor to the disease was proposed to connect hyperglycemia to diabetic damage **[65]** and it was suggested to serve as potential drug target in diabetes **[66, 67]**. Despite considerable evidence showing beneficial effects of antioxidants, results from large-scale clinical trials conclude that classic antioxidant scavengers may not be appropriate therapeutics in diabetes **[68]**.

Mitochondria are involved in the hyperglycemia-induced ROS production both as a source of oxidants and also as an activator of other sources of ROS generation in the cells **[65]**. Glucose uptake occurs via facilitative transporters, thus high extracellular glucose concentration induces a similar rise in the intracellular glucose level. This leads to changes in the substrate availability for cellular metabolism and energy production **[69]** and it also induces ROS production in endothelial cells **[28, 70]**. The mitochondrial oxidative phosphorylation couples substrate oxidation to energy production, during which a low percentage (1–2%) of oxygen is used for superoxide production **[71]**. The main sources of oxidants are complex III and complex I in the mitochondria **[72]**, but since the respiratory complexes form supercomplexes **[73]** and there are changes in the relative amount of the respective respiratory complexes in diabetes **[74–76]**, other complexes may also contribute to the oxidant production. The electrons used for ROS production will leave behind protons in the intermembrane space that may be released via the uncoupling proteins (UCP) to maintain the physiological membrane potential **[77]**. This amount of leak is relatively low compared to the measured ATP production/oxygen atom use (P/O) ratios that estimate approximately 20% basal leakage in the cells **[77]**, but it also requires the neutralization of the oxidants and electron source to resupply the lost ones in the respiratory chain. The mitochondrial antioxidant defense system **[78]** can cope with the basal mitochondrial ROS production and the enzymes involved in the antioxidant defense are upregulated in diabetes **[79]**, but for some reason the oxidant production is not properly counterbalanced in diabetes **[80]**.

Mitochondria produce higher levels of oxidants if the mitochondrial potential is elevated **[81, 82]**. In the b.End3 endothelial cells the glucose-induced ROS production depends on large part on the mitochondrial membrane potential, since there is no change in the ratios of individual mitochondrial respiratory complexes (**Fig 1**) and restoration of the mitochondrial potential inhibits the hyperglycemia-induced mitochondrial superoxide production (**Figs 2–4**). Here we identify UCP2 upregulation as the mode of action of glucocorticoid steroids (**Fig 5**) that emerged as potent inhibitors of the mitochondrial oxidant production in our phenotypic assay **[28]**. Furthermore, UCP2 seems to function as a major controller of mitochondrial ROS production in microvascular endothelial cells, since UCP2 silencing by itself increases the ROS generation and the mitochondrial membrane potential (**Fig 7**).

The primary function of UCP2 and UCP3 (uncoupling protein 3) is the limitation of free radical production in the cells **[83]**. Oxidative stress can rapidly regulate the function of UCP2 via glutathionylation **[84, 85]**: ROS production activates UCP2 that transfers protons back to the mitochondrial matrix and reduces the membrane potential (protects against hyperpolarization), thus it will reduce the ROS generation in the mitochondria, while low oxidant production inactivates UCP2 **[85]**. But the function of the uncoupling proteins is not only controlled at the posttranslational level, transcriptional **[86–90]** and translational **[41, 91]** control of the protein expression was also described. Regulation at this level seems to help adapt the cells to long-term stress. Thus hyperglycemia itself induces UCP2 expression in some cell types, eg. in beta cells **[92]** and venous endothelial cells (**S1 Fig) [93]** and we also observed increased expression level in the muscle in diabetic mice (**S4 Fig**). Consistently, we found that the glucose-induced mitochondrial ROS production is modest in the EA.hy926 venous endothelial cells **[28]** compared to the microvascular endothelial cells, in which hyperglycemia does not alter the expression of UCP2 (**Fig 1**).

Modulation of UCP2 was recommended in diabetes complications **[94]**, since the induction of UCP2 expression is safe: the function of the protein is further controlled at the posttranslational level and it also occurs as an endogenous protective mechanism. Our results confirm that upregulation of UCP2 expression confers benefit against glucose-induced mitochondrial ROS production (**Figs 2–4 and 8**) in microvascular endothelial cells. The functional benefit of uncoupling protein induction in diabetes is also supported by the finding that overexpression of UCP3 (that functions similarly to UCP2 but shows distinct expression profile) is protective against glucose-induced damage in neurons **[95]**. However, the regulation of UCP2 expression is a complex and incompletely understood process and there is no pharmacological tool available to upregulate UCP2 expression. At the RNA level UCP2 expression is affected by micro RNAs (miRNA) in the 3’ untranslated region of the gene: they either stabilize or destabilize the UCP2 transcript upon binding **[90]**. The expression of UCP2 can also be induced via removing the heterogeneous nuclear ribonucleoprotein-K (hnRNP-K) mediated suppression as it was shown for angiopoietin-1 in endothelial cells **[96]**. This mode of action is believed to act at the posttranscriptional level (though the mRNA level of UCP2 was not measured) and also involves phosphorylation by Src tyrosine kinase, a target of glucocorticoid steroids **[97]**. We found that glucocorticoids increase the mRNA level of UCP2 (**Figs 4 and 5**), thus we suspect that the regulation occurs via the above mechanisms, especially, since they could explain the cell-type restricted action of steroids. The microRNA expression profile of the microvascular endothelial cells differ form the macrovascular aortic or venous cells **[98]**, and glucocorticoids induce robust changes in the miRNA expression in the cells **[99–101].** Also, the heterogeneous nuclear ribonucleoprotein subtypes show cell-or tissue specific expression pattern **[102]**. Interestingly, dexamethasone was reported to induce UCP3 expression in C2C12 muscle cells **[40]** via a glucocorticoid response element (GRE) located in the promoter region of the UCP3 gene. But, this mechanism is unlikely to be involved in the UCP2 gene expression, since its promoter region does not contain such sites and the glucocorticoid receptor (GR)-mediated actions occur similarly in most cell types, so the induction would not be cell-type specific. At the translational level, UCP2 expression was reported to be affected by glutamine **[41]** and we found that availability of this amino acid can stimulate the expression of UCP2 in endothelial cells and promote its antioxidant effect (**Fig 6**) if steroids induced the expression at the mRNA level.

## Conclusion

We conclude that UCP2 induction may represent a novel experimental therapeutic intervention in diabetic vascular complications. Direct repurposing of glucocorticoid steroids may not be possible due to their significant side effects that develop during chronic administration, but the UCP2–inducing pathway is may be amenable to upregulation by other pharmacological means that do not affect the glucocorticoid receptors. Additionally, the short-term use of the glucocorticoid receptor antagonist mifepristone may be considered during short hyperglycemic episodes. We expect that specific pharmacological tools that induce UCP2 expression in the microvasculature will be shortly available and that will allow the translation of this concept to clinical practice.

## Supporting Information

S1 Fig High-glucose induced UCP2 expression in EA.hy926 cells. **A, B:** EA.hy926 human venous endothelial cells were exposed to high glucose for 7 days and treated with dexamethasone (1 μM) for 3 days. UCP2 expression was determined by Western blotting. Representative blot image (**A**) and densitometric analysis results (**B**) are shown. (#p<0.05 high-glucose exposure induced significant increase in UCP2 expression,*p<0.05 dexamethasone treatment significantly reduced the UCP2 expression.)
(TIFF)

S2 Fig High-glucose induced decrease in mitochondrial DNA copy number. b.End3 cells were exposed to high glucose for 7 days and treated subsequently with dexamethasone (1μM) for 3 days. DNA was isolated form the cells and relative amount of mitochondrial and genomic DNA was determined by Taqman assay. (#p<0.05 high-glucose exposure induced significant changes compared to cells maintained in low glucose containing medium,*p<0.05 dexamethasone treatment significantly reduced the mitochondrial DNA content.)
(TIFF)

S3 Fig Dexamethasone-induced gene expression changes. **A-E:** b.End3 cells were exposed to high glucose for 7 days and treated subsequently with dexamethasone (1 μM) for 3 days. Relative gene expression was determined by realtime PCR and normalized to 18S rRNA levels. The expression of **A**: the glucocorticoid receptor (GR), **B**: sirtuin 1 (SIRT), **C**: the mitochondrial 16S rRNA (16S RNA), **D**: cytochrome C (Cyt C) and **E**: the cytochrome C oxidase subunit 3 (COX3) were determined. (*p<0.05 dexamethasone induced significant changes in gene expression.)
(TIFF)

S4 Fig UCP2 upregulation in diabetic muscle. Diabetes was induced in 8-week old NMRI male mice (Charles River) by multiple low-dose streptozotocin (50 mg/kg on 5 consecutive days) protocol. Hyperglycemia was confirmed 2 weeks following the streptozotocin injections and muscle samples were collected. Gene expression was measured by UCP2 (assay ID: Mm00627598, Life Technologies) Taqman assay in combination with the normalization signal (VIC-labeled 18S rRNA control reagents, Cat# 438329, Life Technologies). Normalized expression values are shown. (*p<0.05 Diabetes induced significant increase in UCP2 expression, n = 6/group.)
(TIFF)

S1 File Supplementary methods. (DOC)

## References

[1] Fowler MJ. Microvascular and Macrovascular Complications of Diabetes. Clinical Diabetes. 2008;26(2):77–82. 10.2337/diaclin.26.2.77

[2] Intensive blood-glucose control with sulphonylureas or insulin compared with conventional treatment and risk of complications in patients with type 2 diabetes (UKPDS 33). UK Prospective Diabetes Study (UKPDS) Group. Lancet. 1998;352(9131):837–53. . 9742976

[3] Mannucci E, Dicembrini I, Lauria A, Pozzilli P. Is glucose control important for prevention of cardiovascular disease in diabetes? Diabetes care. 2013;36 Suppl 2:S259–63. 10.2337/dcS13-2018 23882055 PMC3920786

[4] Singh PP, Mahadi F, Roy A, Sharma P. Reactive oxygen species, reactive nitrogen species and antioxidants in etiopathogenesis of diabetes mellitus type-2. Indian J Clin Biochem. 2009;24(4):324–42. Epub 2009/10/01. 10.1007/s12291-009-0062-662 [pii]. 23105858 PMC3453064

[5] Ceriello A. New insights on oxidative stress and diabetic complications may lead to a "causal" antioxidant therapy. Diabetes care. 2003;26(5):1589–96. Epub 2003/04/30. . 12716823 10.2337/diacare.26.5.1589

[6] Ceriello A. Diabetic complications: from oxidative stress to inflammatory cardiovascular disorders. Medicographia. 2011;33(1):29–34.

[7] Giacco F, Brownlee M. Oxidative stress and diabetic complications. Circ Res. 2011;107(9):1058–70. Epub 2010/10/30. 107/9/1058 [pii] 10.1161/CIRCRESAHA.110.223545 21030723 PMC2996922

[8] Giugliano D, Ceriello A, Paolisso G. Oxidative stress and diabetic vascular complications. Diabetes care. 1996;19(3):257–67. Epub 1996/03/01. . 8742574 10.2337/diacare.19.3.257

[9] Huerta MG, Nadler JL. Oxidative stress, inflammation and diabetic complications In: LeRoith D, Olefsky JM, Taylor SI, editors. Diabetes Mellitus: A Fundamental and Clinical Text: Lippincott Williams & Wilkins (LWW); 2003 p. 1485–502.

[10] Bowry VW, Ingold KU, Stocker R. Vitamin E in human low-density lipoprotein. When and how this antioxidant becomes a pro-oxidant. Biochem J. 1992;288 (Pt 2):341–4. Epub 1992/12/01. 1463440 10.1042/bj2880341 PMC1132016

[11] Hunt JV, Bottoms MA, Mitchinson MJ. Ascorbic acid oxidation: a potential cause of the elevated severity of atherosclerosis in diabetes mellitus? FEBS Lett. 1992;311(2):161–4. Epub 1992/10/19. 0014-5793(92)81389-4 [pii]. . 1397304 10.1016/0014-5793(92)81389-4

[12] Young IS, Tate S, Lightbody JH, McMaster D, Trimble ER. The effects of desferrioxamine and ascorbate on oxidative stress in the streptozotocin diabetic rat. Free Radic Biol Med. 1995;18(5):833–40. Epub 1995/05/01. 089158499400202U [pii]. . 7797090 10.1016/0891-5849(94)00202-u

[13] Tucker S, Ahl M, Bush A, Westaway D, Huang X, Rogers JT. Pilot study of the reducing effect on amyloidosis in vivo by three FDA pre-approved drugs via the Alzheimer's APP 5' untranslated region. Curr Alzheimer Res. 2005;2(2):249–54. Epub 2005/06/25. . 15974925 10.2174/1567205053585855

[14] Meng B, Li J, Cao H. Antioxidant and antiinflammatory activities of curcumin on diabetes mellitus and its complications. Curr Pharm Des. 2012;19(11):2101–13. Epub 2012/11/03. CPD-EPUB-20121023-12 [pii]. . 23116316

[15] Kuhad A, Chopra K. Effect of sesamol on diabetes-associated cognitive decline in rats. Exp Brain Res. 2008;185(3):411–20. Epub 2007/10/24. 10.1007/s00221-007-1166-y . 17955223

[16] Christ M, Bauersachs J, Liebetrau C, Heck M, Gunther A, Wehling M. Glucose increases endothelial-dependent superoxide formation in coronary arteries by NAD(P)H oxidase activation: attenuation by the 3-hydroxy-3-methylglutaryl coenzyme A reductase inhibitor atorvastatin. Diabetes. 2002;51(8):2648–52. Epub 2002/07/30. . 12145183 10.2337/diabetes.51.8.2648

[17] Dorenkamp M, Riad A, Stiehl S, Spillmann F, Westermann D, Du J, et al Protection against oxidative stress in diabetic rats: role of angiotensin AT(1) receptor and beta 1-adrenoceptor antagonism. Eur J Pharmacol. 2005;520(1–3):179–87. Epub 2005/09/06. S0014-2999(05)00753-3 [pii] 10.1016/j.ejphar.2005.07.020 . 16139267

[18] Tawfik HE, El-Remessy AB, Matragoon S, Ma G, Caldwell RB, Caldwell RW. Simvastatin improves diabetes-induced coronary endothelial dysfunction. J Pharmacol Exp Ther. 2006;319(1):386–95. Epub 2006/07/20. jpet.106.106823 [pii] 10.1124/jpet.106.106823 . 16849625

[19] Vecchione C, Aretini A, Marino G, Bettarini U, Poulet R, Maffei A, et al Selective Rac-1 inhibition protects from diabetes-induced vascular injury. Circ Res. 2006;98(2):218–25. Epub 2005/12/17. 01.RES.0000200440.18768.30 [pii] 10.1161/01.RES.0000200440.18768.30 . 16357302

[20] Hwang J, Kleinhenz DJ, Rupnow HL, Campbell AG, Thule PM, Sutliff RL, et al The PPARgamma ligand, rosiglitazone, reduces vascular oxidative stress and NADPH oxidase expression in diabetic mice. Vascul Pharmacol. 2007;46(6):456–62. Epub 2007/03/06. S1537-1891(07)00033-X [pii] 10.1016/j.vph.2007.01.007 . 17337254

[21] Piconi L, Corgnali M, Da Ros R, Assaloni R, Piliego T, Ceriello A. The protective effect of rosuvastatin in human umbilical endothelial cells exposed to constant or intermittent high glucose. J Diabetes Complications. 2008;22(1):38–45. Epub 2008/01/15. S1056-8727(07)00039-6 [pii] 10.1016/j.jdiacomp.2007.03.004 . 18191076

[22] Van Linthout S, Spillmann F, Lorenz M, Meloni M, Jacobs F, Egorova M, et al Vascular-protective effects of high-density lipoprotein include the downregulation of the angiotensin II type 1 receptor. Hypertension. 2009;53(4):682–7. Epub 2009/03/11. HYPERTENSIONAHA.108.118919 [pii] 10.1161/HYPERTENSIONAHA.108.118919 . 19273745

[23] Li G, Tang J, Du Y, Lee CA, Kern TS. Beneficial effects of a novel RAGE inhibitor on early diabetic retinopathy and tactile allodynia. Mol Vis. 2011;17:3156–65. Epub 2011/12/16. 340 [pii]. 22171162 PMC3235538

[24] Szabo C, Biser A, Benko R, Bottinger E, Susztak K. Poly(ADP-ribose) polymerase inhibitors ameliorate nephropathy of type 2 diabetic Leprdb/db mice. Diabetes. 2006;55(11):3004–12. Epub 2006/10/27. 55/11/3004 [pii] 10.2337/db06-0147 . 17065336

[25] Calcutt NA, Cooper ME, Kern TS, Schmidt AM. Therapies for hyperglycaemia-induced diabetic complications: from animal models to clinical trials. Nat Rev Drug Discov. 2009;8(5):417–29. Epub 2009/05/01. nrd2476 [pii] 10.1038/nrd2476 . 19404313 PMC7097138

[26] Yamagishi S, Maeda S, Matsui T, Ueda S, Fukami K, Okuda S. Role of advanced glycation end products (AGEs) and oxidative stress in vascular complications in diabetes. Biochim Biophys Acta. 2011;1820(5):663–71. Epub 2011/03/29. S0304-4165(11)00063-8 [pii] 10.1016/j.bbagen.2011.03.014 . 21440603

[27] Nishikawa T, Edelstein D, Du XL, Yamagishi S, Matsumura T, Kaneda Y, et al Normalizing mitochondrial superoxide production blocks three pathways of hyperglycaemic damage. Nature. 2000;404(6779):787–90. Epub 2000/04/28. 10.1038/35008121 . 10783895

[28] Gero D, Szoleczky P, Suzuki K, Modis K, Olah G, Coletta C, et al Cell-based screening identifies paroxetine as an inhibitor of diabetic endothelial dysfunction. Diabetes. 2013;62(3):953–64. Epub 2012/12/12. db12-0789 [pii] 10.2337/db12-0789 23223176 PMC3581231

[29] Williams RL, Risau W, Zerwes HG, Drexler H, Aguzzi A, Wagner EF. Endothelioma cells expressing the polyoma middle T oncogene induce hemangiomas by host cell recruitment. Cell. 1989;57(6):1053–63. . 2736622 10.1016/0092-8674(89)90343-7

[30] Montesano R, Pepper MS, Mohle-Steinlein U, Risau W, Wagner EF, Orci L. Increased proteolytic activity is responsible for the aberrant morphogenetic behavior of endothelial cells expressing the middle T oncogene. Cell. 1990;62(3):435–45. . 2379237 10.1016/0092-8674(90)90009-4

[31] Sikorski EE, Hallmann R, Berg EL, Butcher EC. The Peyer's patch high endothelial receptor for lymphocytes, the mucosal vascular addressin, is induced on a murine endothelial cell line by tumor necrosis factor-alpha and IL-1. Journal of immunology. 1993;151(10):5239–50. . 7693807

[32] Gero D, Szabo C. Nicotinamide adenine dinucleotide (NAD) salvage plays critical role in bioenergetic recovery of posthypoxic cardiomyocytes. British journal of pharmacology. 2015. 10.1111/bph.13252 PMC4621988 26218637

[33] Keij JF, Bell-Prince C, Steinkamp JA. Staining of mitochondrial membranes with 10-nonyl acridine orange, MitoFluor Green, and MitoTracker Green is affected by mitochondrial membrane potential altering drugs. Cytometry. 2000;39(3):203–10. . 10685077 10.1002/(sici)1097-0320(20000301)39:3<203::aid-cyto5>3.0.co;2-z

[34] Gero D, Modis K, Nagy N, Szoleczky P, Toth ZD, Dorman G, et al Oxidant-induced cardiomyocyte injury: identification of the cytoprotective effect of a dopamine 1 receptor agonist using a cell-based high-throughput assay. International journal of molecular medicine. 2007;20(5):749–61. . 17912470

[35] Gero D, Szoleczky P, Modis K, Pribis JP, Al-Abed Y, Yang H, et al Identification of pharmacological modulators of HMGB1-induced inflammatory response by cell-based screening. PLoS One. 2013;8(6):e65994 10.1371/journal.pone.0065994 23799067 PMC3682954

[36] Gero D, Szoleczky P, Chatzianastasiou A, Papapetropoulos A, Szabo C. Modulation of poly(ADP-ribose) polymerase-1 (PARP-1)-mediated oxidative cell injury by ring finger protein 146 (RNF146) in cardiac myocytes. Molecular medicine. 2014;20:313–28. 10.2119/molmed.2014.00102 24842055 PMC4153837

[37] Szoleczky P, Modis K, Nagy N, Dori Toth Z, DeWitt D, Szabo C, et al Identification of agents that reduce renal hypoxia-reoxygenation injury using cell-based screening: purine nucleosides are alternative energy sources in LLC-PK1 cells during hypoxia. Archives of biochemistry and biophysics. 2012;517(1):53–70. 10.1016/j.abb.2011.11.005 . 22100704 PMC4676579

[38] Modis K, Gero D, Erdelyi K, Szoleczky P, DeWitt D, Szabo C. Cellular bioenergetics is regulated by PARP1 under resting conditions and during oxidative stress. Biochemical pharmacology. 2012;83(5):633–43. 10.1016/j.bcp.2011.12.014 22198485 PMC3272837

[39] Supino R. MTT assays. Methods in molecular biology. 1995;43:137–49. 10.1385/0-89603-282-5:137 . 7550641

[40] Amat R, Solanes G, Giralt M, Villarroya F. SIRT1 is involved in glucocorticoid-mediated control of uncoupling protein-3 gene transcription. The Journal of biological chemistry. 2007;282(47):34066–76. 10.1074/jbc.M707114200 . 17884810

[41] Hurtaud C, Gelly C, Chen Z, Levi-Meyrueis C, Bouillaud F. Glutamine stimulates translation of uncoupling protein 2mRNA. Cellular and molecular life sciences: CMLS. 2007;64(14):1853–60. 10.1007/s00018-007-7039-5 . 17514359 PMC11136301

[42] Nau PN, Van Natta T, Ralphe JC, Teneyck CJ, Bedell KA, Caldarone CA, et al Metabolic adaptation of the fetal and postnatal ovine heart: regulatory role of hypoxia-inducible factors and nuclear respiratory factor-1. Pediatric research. 2002;52(2):269–78. 10.1203/00006450-200208000-00021 . 12149506

[43] Marone JR, Falduto MT, Essig DA, Hickson RC. Effects of glucocorticoids and endurance training on cytochrome oxidase expression in skeletal muscle. Journal of applied physiology. 1994;77(4):1685–90. . 7836187 10.1152/jappl.1994.77.4.1685

[44] Rachamim N, Latter H, Malinin N, Asher C, Wald H, Garty H. Dexamethasone enhances expression of mitochondrial oxidative phosphorylation genes in rat distal colon. The American journal of physiology. 1995;269(5 Pt 1):C1305–10. . 7491922 10.1152/ajpcell.1995.269.5.C1305

[45] Weber K, Bruck P, Mikes Z, Kupper JH, Klingenspor M, Wiesner RJ. Glucocorticoid hormone stimulates mitochondrial biogenesis specifically in skeletal muscle. Endocrinology. 2002;143(1):177–84. 10.1210/endo.143.1.8600 . 11751607

[46] Scheller K, Sekeris CE. The effects of steroid hormones on the transcription of genes encoding enzymes of oxidative phosphorylation. Experimental physiology. 2003;88(1):129–40. . 12525861 10.1113/eph8802507

[47] Psarra AM, Sekeris CE. Glucocorticoids induce mitochondrial gene transcription in HepG2 cells: role of the mitochondrial glucocorticoid receptor. Biochim Biophys Acta. 2011;1813(10):1814–21. 10.1016/j.bbamcr.2011.05.014 . 21664385

[48] Minchenko AG. [Role of glucocorticoids in the mitochondrial DNA replication]. Problemy endokrinologii. 1979;25(5):52–6. . 493255

[49] Lee SR, Kim HK, Song IS, Youm J, Dizon LA, Jeong SH, et al Glucocorticoids and their receptors: insights into specific roles in mitochondria. Progress in biophysics and molecular biology. 2013;112(1–2):44–54. 10.1016/j.pbiomolbio.2013.04.001 . 23603102

[50] Stankov MV, Lucke T, Das AM, Schmidt RE, Behrens GM. Mitochondrial DNA depletion and respiratory chain activity in primary human subcutaneous adipocytes treated with nucleoside analogue reverse transcriptase inhibitors. Antimicrobial agents and chemotherapy. 2010;54(1):280–7. 10.1128/AAC.00914-09 19805555 PMC2798495

[51] Reinecke F, Smeitink JA, van der Westhuizen FH. OXPHOS gene expression and control in mitochondrial disorders. Biochim Biophys Acta. 2009;1792(12):1113–21. 10.1016/j.bbadis.2009.04.003 . 19389473

[52] Vempati UD, Han X, Moraes CT. Lack of cytochrome c in mouse fibroblasts disrupts assembly/stability of respiratory complexes I and IV. The Journal of biological chemistry. 2009;284(7):4383–91. 10.1074/jbc.M805972200 19075019 PMC2640958

[53] Li K, Li Y, Shelton JM, Richardson JA, Spencer E, Chen ZJ, et al Cytochrome c deficiency causes embryonic lethality and attenuates stress-induced apoptosis. Cell. 2000;101(4):389–99. . 10830166 10.1016/s0092-8674(00)80849-1

[54] Zhang J, Khvorostov I, Hong JS, Oktay Y, Vergnes L, Nuebel E, et al UCP2 regulates energy metabolism and differentiation potential of human pluripotent stem cells. The EMBO journal. 2011;30(24):4860–73. 10.1038/emboj.2011.401 22085932 PMC3243621

[55] Ohkouchi S, Block GJ, Katsha AM, Kanehira M, Ebina M, Kikuchi T, et al Mesenchymal stromal cells protect cancer cells from ROS-induced apoptosis and enhance the Warburg effect by secreting STC1. Molecular therapy: the journal of the American Society of Gene Therapy. 2012;20(2):417–23. 10.1038/mt.2011.259 22146344 PMC3277221

[56] World Health Organization., International Diabetes Federation. Definition and diagnosis of diabetes mellitus and intermediate hyperglycaemia: report of a WHO/IDF consultation Geneva: World Health Organization. p. p.

[57] Standards of medical care in diabetes—2015: summary of revisions. Diabetes care. 2015;38 Suppl:S4 10.2337/dc15-S003 . 25537706

[58] Alberti KG, Zimmet PZ. Definition, diagnosis and classification of diabetes mellitus and its complications. Part 1: diagnosis and classification of diabetes mellitus provisional report of a WHO consultation [see comments]1998. 10.1002/(SICI)1096-9136(199807)15:7<539::AID-DIA668>3.0.CO;2-S 9686693

[59] World Health Organization. Dept. of Noncommunicable Disease Surveillance. Definition, diagnosis and classification of diabetes mellitus and its complications: report of a WHO consultation Part 1, Diagnosis and classification of diabetes mellitus. Geneva: World Health Organization; 1999. 59 p. p.

[60] Brown SH, Abdelhafiz AH. Trials review: cardiovascular outcome with intensive glycemic control and implications for patients with type 2 diabetes. Postgraduate medicine. 2009;121(5):31–41. 10.3810/pgm.2009.09.2050 . 19820272

[61] Cheng AY, Leiter LA. Glucose lowering and cardiovascular disease: what do we know and what should we do? European journal of cardiovascular prevention and rehabilitation: official journal of the European Society of Cardiology, Working Groups on Epidemiology & Prevention and Cardiac Rehabilitation and Exercise Physiology. 2010;17 Suppl 1:S25–31. 10.1097/01.hjr.0000368194.32356.5f . 20489417

[62] Skyler JS. Effects of Glycemic Control on Diabetes Complications and on the Prevention of Diabetes. Clinical Diabetes. 2004;22(4):162–6. 10.2337/diaclin.22.4.162

[63] Action to Control Cardiovascular Risk in Diabetes Study G, Gerstein HC, Miller ME, Byington RP, Goff DC Jr., Bigger JT, et al Effects of intensive glucose lowering in type 2 diabetes. The New England journal of medicine. 2008;358(24):2545–59. 10.1056/NEJMoa0802743 . 18539917 PMC4551392

[64] McAlpine RR, Morris AD, Emslie-Smith A, James P, Evans JM. The annual incidence of diabetic complications in a population of patients with Type 1 and Type 2 diabetes. Diabetic medicine: a journal of the British Diabetic Association. 2005;22(3):348–52. 10.1111/j.1464-5491.2004.01391.x . 15717888

[65] Brownlee M. The pathobiology of diabetic complications: a unifying mechanism. Diabetes. 2005;54(6):1615–25. . 15919781 10.2337/diabetes.54.6.1615

[66] Ceriello A, Testa R. Antioxidant anti-inflammatory treatment in type 2 diabetes. Diabetes care. 2009;32 Suppl 2:S232–6. 10.2337/dc09-S316 19875557 PMC2811469

[67] Wu Y, Tang L, Chen B. Oxidative stress: implications for the development of diabetic retinopathy and antioxidant therapeutic perspectives. Oxidative medicine and cellular longevity. 2014;2014:752387 10.1155/2014/752387 25180070 PMC4142742

[68] Johansen JS, Harris AK, Rychly DJ, Ergul A. Oxidative stress and the use of antioxidants in diabetes: linking basic science to clinical practice. Cardiovascular diabetology. 2005;4:5 10.1186/1475-2840-4-5 15862133 PMC1131912

[69] Lu J, Xie G, Jia W, Jia W. Metabolomics in human type 2 diabetes research. Frontiers of medicine. 2013;7(1):4–13. 10.1007/s11684-013-0248-4 . 23377891

[70] Quijano C, Castro L, Peluffo G, Valez V, Radi R. Enhanced mitochondrial superoxide in hyperglycemic endothelial cells: direct measurements and formation of hydrogen peroxide and peroxynitrite. American journal of physiology Heart and circulatory physiology. 2007;293(6):H3404–14. 10.1152/ajpheart.00761.2007 . 17906108

[71] Turrens JF. Mitochondrial formation of reactive oxygen species. The Journal of physiology. 2003;552(Pt 2):335–44. 10.1113/jphysiol.2003.049478 14561818 PMC2343396

[72] Lenaz G. The mitochondrial production of reactive oxygen species: mechanisms and implications in human pathology. IUBMB life. 2001;52(3–5):159–64. 10.1080/15216540152845957 . 11798028

[73] Lenaz G, Baracca A, Barbero G, Bergamini C, Dalmonte ME, Del Sole M, et al Mitochondrial respiratory chain super-complex I-III in physiology and pathology. Biochim Biophys Acta. 2010;1797(6–7):633–40. 10.1016/j.bbabio.2010.01.025 . 20116362

[74] Ferreira FM, Palmeira CM, Seica R, Moreno AJ, Santos MS. Diabetes and mitochondrial bioenergetics: alterations with age. Journal of biochemical and molecular toxicology. 2003;17(4):214–22. 10.1002/jbt.10081 . 12898645

[75] Munusamy S, Saba H, Mitchell T, Megyesi JK, Brock RW, Macmillan-Crow LA. Alteration of renal respiratory Complex-III during experimental type-1 diabetes. BMC endocrine disorders. 2009;9:2 10.1186/1472-6823-9-2 19166612 PMC2636815

[76] Shenouda SM, Widlansky ME, Chen K, Xu G, Holbrook M, Tabit CE, et al Altered mitochondrial dynamics contributes to endothelial dysfunction in diabetes mellitus. Circulation. 2011;124(4):444–53. 10.1161/CIRCULATIONAHA.110.014506 21747057 PMC3149100

[77] Brand MD. The efficiency and plasticity of mitochondrial energy transduction. Biochemical Society transactions. 2005;33(Pt 5):897–904. 10.1042/BST20050897 . 16246006

[78] Rabilloud T, Heller M, Rigobello MP, Bindoli A, Aebersold R, Lunardi J. The mitochondrial antioxidant defence system and its response to oxidative stress. Proteomics. 2001;1(9):1105–10. 10.1002/1615-9861(200109)1:9<1105::AID-PROT1105>3.0.CO;2-M . 11990504

[79] Celik VK, Sahin ZD, Sari I, Bakir S. Comparison of oxidant/antioxidant, detoxification systems in various tissue homogenates and mitochondria of rats with diabetes induced by streptozocin. Experimental diabetes research. 2012;2012:386831 10.1155/2012/386831 22536214 PMC3320003

[80] Sharma K. Mitochondrial hormesis and diabetic complications. Diabetes. 2015;64(3):663–72. 10.2337/db14-0874 25713188 PMC4338592

[81] Korshunov SS, Skulachev VP, Starkov AA. High protonic potential actuates a mechanism of production of reactive oxygen species in mitochondria. FEBS Lett. 1997;416(1):15–8. . 9369223 10.1016/s0014-5793(97)01159-9

[82] Suski JM, Lebiedzinska M, Bonora M, Pinton P, Duszynski J, WM R.. Relation Between Mitochondrial Membrane Potential and ROS Formation In: Palmeira CMaMAJ, editor. Mitochondrial Bioenergetics: Methods and Protocols Methods in Molecular Biology. 810: Springer Science+Business Media, LLC; 2012 p. 183–205. 10.1007/978-1-61779-382-0_12 22057568

[83] Rousset S, Alves-Guerra MC, Mozo J, Miroux B, Cassard-Doulcier AM, Bouillaud F, et al The biology of mitochondrial uncoupling proteins. Diabetes. 2004;53 Suppl 1:S130–5. . 14749278 10.2337/diabetes.53.2007.s130

[84] Mailloux RJ, Harper ME. Uncoupling proteins and the control of mitochondrial reactive oxygen species production. Free Radic Biol Med. 2011;51(6):1106–15. 10.1016/j.freeradbiomed.2011.06.022 . 21762777

[85] Mailloux RJ, Seifert EL, Bouillaud F, Aguer C, Collins S, Harper ME. Glutathionylation acts as a control switch for uncoupling proteins UCP2 and UCP3. The Journal of biological chemistry. 2011;286(24):21865–75. 10.1074/jbc.M111.240242 21515686 PMC3122241

[86] Martin-Oliva D, Aguilar-Quesada R, O'Valle F, Munoz-Gamez JA, Martinez-Romero R, Garcia Del Moral R, et al Inhibition of poly(ADP-ribose) polymerase modulates tumor-related gene expression, including hypoxia-inducible factor-1 activation, during skin carcinogenesis. Cancer research. 2006;66(11):5744–56. 10.1158/0008-5472.CAN-05-3050 . 16740713

[87] Berraondo B, Marti A, Duncan JS, Trayhurn P, Martinez JA. Up-regulation of muscle UCP2 gene expression by a new beta3-adrenoceptor agonist, trecadrine, in obese (cafeteria) rodents, but down-regulation in lean animals. International journal of obesity and related metabolic disorders: journal of the International Association for the Study of Obesity. 2000;24(2):156–63. . 10702765 10.1038/sj.ijo.0801097

[88] Chan SH, Wu CA, Wu KL, Ho YH, Chang AY, Chan JY. Transcriptional upregulation of mitochondrial uncoupling protein 2 protects against oxidative stress-associated neurogenic hypertension. Circ Res. 2009;105(9):886–96. 10.1161/CIRCRESAHA.109.199018 . 19762685

[89] Chen XL, Tang WX, Tang XH, Qin W, Gong M. Downregulation of uncoupling protein-2 by genipin exacerbates diabetes-induced kidney proximal tubular cells apoptosis. Renal failure. 2014;36(8):1298–303. 10.3109/0886022X.2014.930650 . 24964191

[90] Chen X, Wang K, Chen J, Guo J, Yin Y, Cai X, et al In vitro evidence suggests that miR-133a-mediated regulation of uncoupling protein 2 (UCP2) is an indispensable step in myogenic differentiation. The Journal of biological chemistry. 2009;284(8):5362–9. 10.1074/jbc.M807523200 . 19073597

[91] Pecqueur C, Alves-Guerra MC, Gelly C, Levi-Meyrueis C, Couplan E, Collins S, et al Uncoupling protein 2, in vivo distribution, induction upon oxidative stress, and evidence for translational regulation. The Journal of biological chemistry. 2001;276(12):8705–12. 10.1074/jbc.M006938200 . 11098051

[92] Chan CB, Saleh MC, Koshkin V, Wheeler MB. Uncoupling protein 2 and islet function. Diabetes. 2004;53 Suppl 1:S136–42. . 14749279 10.2337/diabetes.53.2007.s136

[93] Koziel A, Woyda-Ploszczyca A, Kicinska A, Jarmuszkiewicz W. The influence of high glucose on the aerobic metabolism of endothelial EA.hy926 cells. Pflugers Archiv: European journal of physiology. 2012;464(6):657–69. 10.1007/s00424-012-1156-1 23053476 PMC3513600

[94] Souza BM, Assmann TS, Kliemann LM, Gross JL, Canani LH, Crispim D. The role of uncoupling protein 2 (UCP2) on the development of type 2 diabetes mellitus and its chronic complications. Arquivos brasileiros de endocrinologia e metabologia. 2011;55(4):239–48. . 21779625 10.1590/s0004-27302011000400001

[95] Vincent AM, Olzmann JA, Brownlee M, Sivitz WI, Russell JW. Uncoupling proteins prevent glucose-induced neuronal oxidative stress and programmed cell death. Diabetes. 2004;53(3):726–34. Epub 2004/02/28. . 14988258 10.2337/diabetes.53.3.726

[96] Tahir TA, Singh H, Brindle NP. The RNA binding protein hnRNP-K mediates post-transcriptional regulation of uncoupling protein-2 by angiopoietin-1. Cellular signalling. 2014;26(7):1379–84. 10.1016/j.cellsig.2014.03.005 24642125 PMC4039131

[97] Yang S, Roselli F, Patchev AV, Yu S, Almeida OF. Non-receptor-tyrosine kinases integrate fast glucocorticoid signaling in hippocampal neurons. The Journal of biological chemistry. 2013;288(33):23725–39. 10.1074/jbc.M113.470146 23818519 PMC3745320

[98] McCall MN, Kent OA, Yu J, Fox-Talbot K, Zaiman AL, Halushka MK. MicroRNA profiling of diverse endothelial cell types. BMC medical genomics. 2011;4:78 10.1186/1755-8794-4-78 22047531 PMC3223144

[99] Hu Z, Shen WJ, Cortez Y, Tang X, Liu LF, Kraemer FB, et al Hormonal regulation of microRNA expression in steroid producing cells of the ovary, testis and adrenal gland. PLoS One. 2013;8(10):e78040 10.1371/journal.pone.0078040 24205079 PMC3810252

[100] Smith LK, Shah RR, Cidlowski JA. Glucocorticoids modulate microRNA expression and processing during lymphocyte apoptosis. The Journal of biological chemistry. 2010;285(47):36698–708. 10.1074/jbc.M110.162123 20847043 PMC2978599

[101] Li T, Li H, Li T, Fan J, Zhao RC, Weng X. MicroRNA expression profile of dexamethasone-induced human bone marrow-derived mesenchymal stem cells during osteogenic differentiation. Journal of cellular biochemistry. 2014;115(10):1683–91. 10.1002/jcb.24831 . 24802236

[102] Kamma H, Portman DS, Dreyfuss G. Cell type-specific expression of hnRNP proteins. Experimental cell research. 1995;221(1):187–96. 10.1006/excr.1995.1366 . 7589244

