# Supplementary figures and images for: Glucocorticoids Suppress Mitochondrial Oxidant Production via Upregulation of Uncoupling Protein 2 in Hyperglycemic Endothelial Cells
Source: PLoS One. 2016 Apr 29;11(4):e0154813. doi: 10.1371/journal.pone.0154813 (PMC4851329; doi:10.1371/journal.pone.0154813)

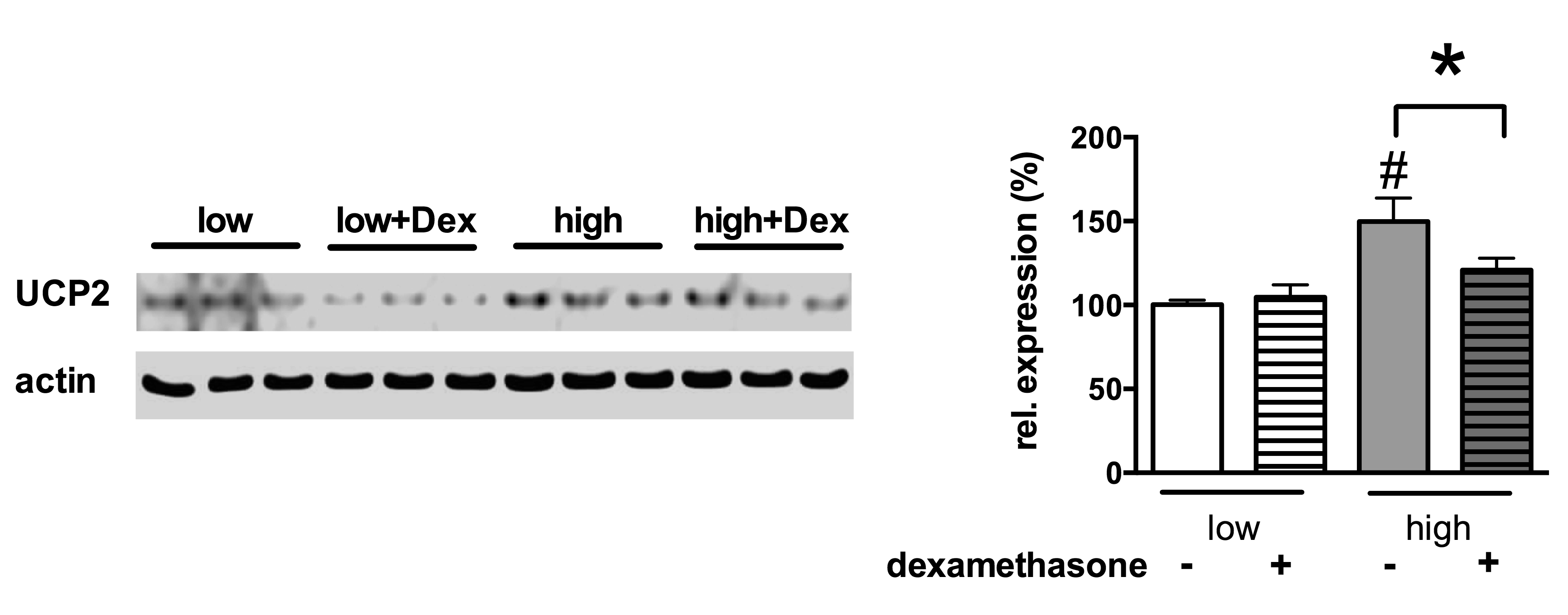

Supplement: S1 Fig — A, B: EA.hy926 human venous endothelial cells were exposed to high glucose for 7 days and treated with dexamethasone (1 μM) for 3 days. UCP2 expression was determined by Western blotting. Representative blot image (A) and densitometric analysis results (B) are shown. (#p<0.05 high-glucose exposure induced significant increase in UCP2 expression,*p<0.05 dexamethasone treatment significantly reduced the UCP2 expression.) (TIFF) [file pone.0154813.s001.tiff]

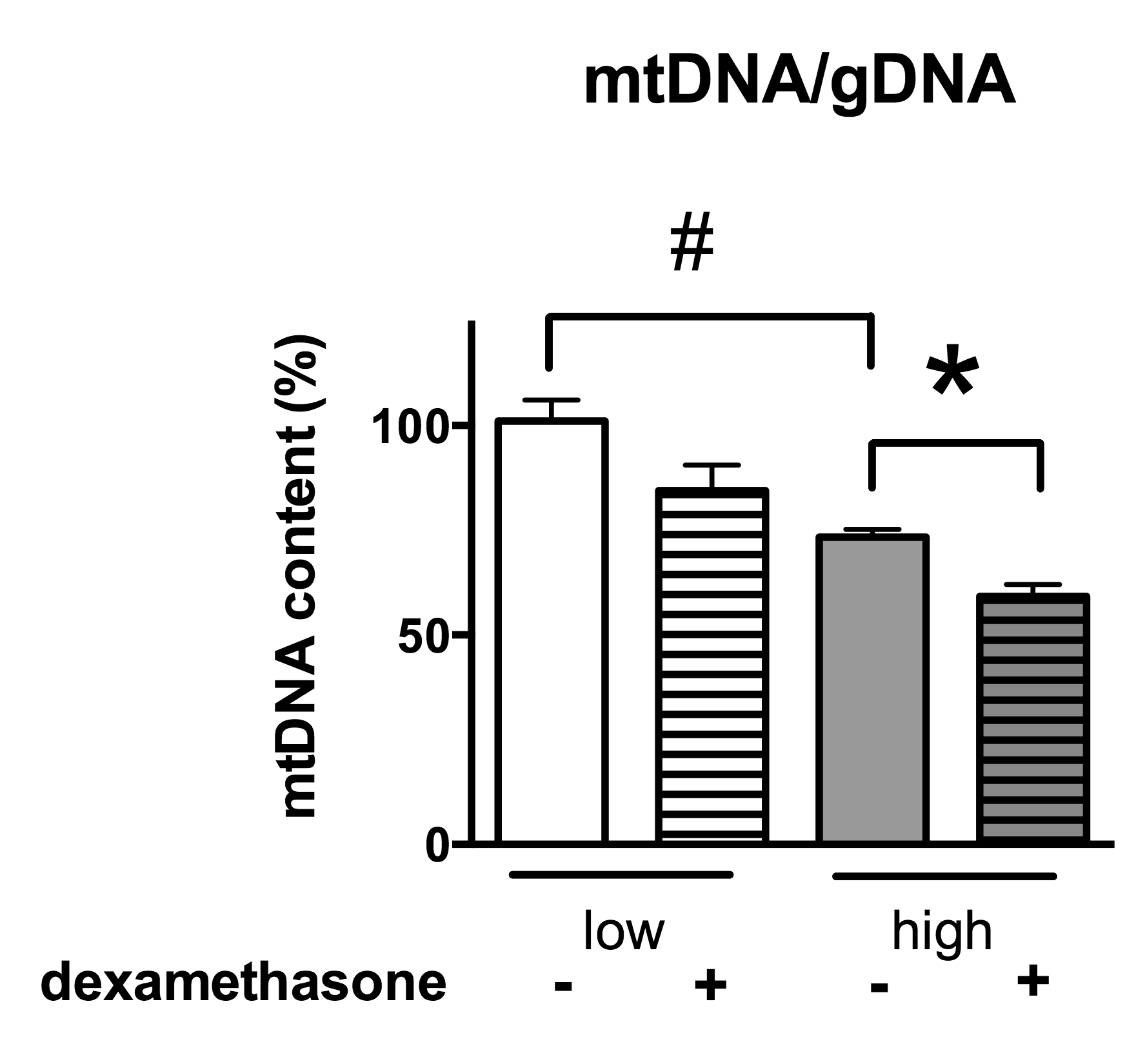

Supplement: S2 Fig — b.End3 cells were exposed to high glucose for 7 days and treated subsequently with dexamethasone (1μM) for 3 days. DNA was isolated form the cells and relative amount of mitochondrial and genomic DNA was determined by Taqman assay. (#p<0.05 high-glucose exposure induced significant changes compared to cells maintained in low glucose containing medium,*p<0.05 dexamethasone treatment significantly reduced the mitochondrial DNA content.) (TIFF) [file pone.0154813.s002.tiff]

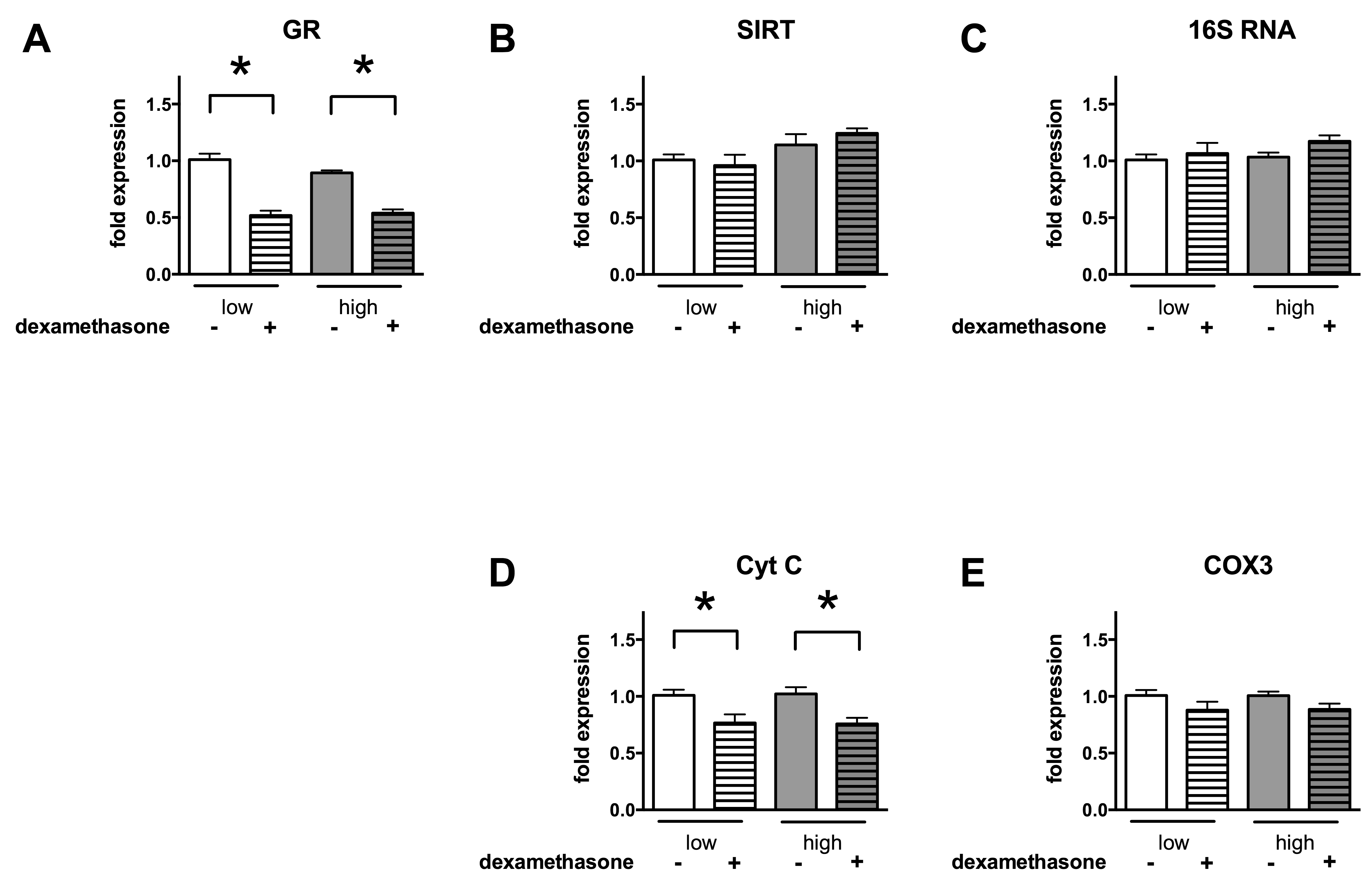

Supplement: S3 Fig — A-E: b.End3 cells were exposed to high glucose for 7 days and treated subsequently with dexamethasone (1 μM) for 3 days. Relative gene expression was determined by realtime PCR and normalized to 18S rRNA levels. The expression of A: the glucocorticoid receptor (GR), B: sirtuin 1 (SIRT), C: the mitochondrial 16S rRNA (16S RNA), D: cytochrome C (Cyt C) and E: the cytochrome C oxidase subunit 3 (COX3) were determined. (*p<0.05 dexamethasone induced significant changes in gene expression.) (TIFF) [file pone.0154813.s003.tiff]

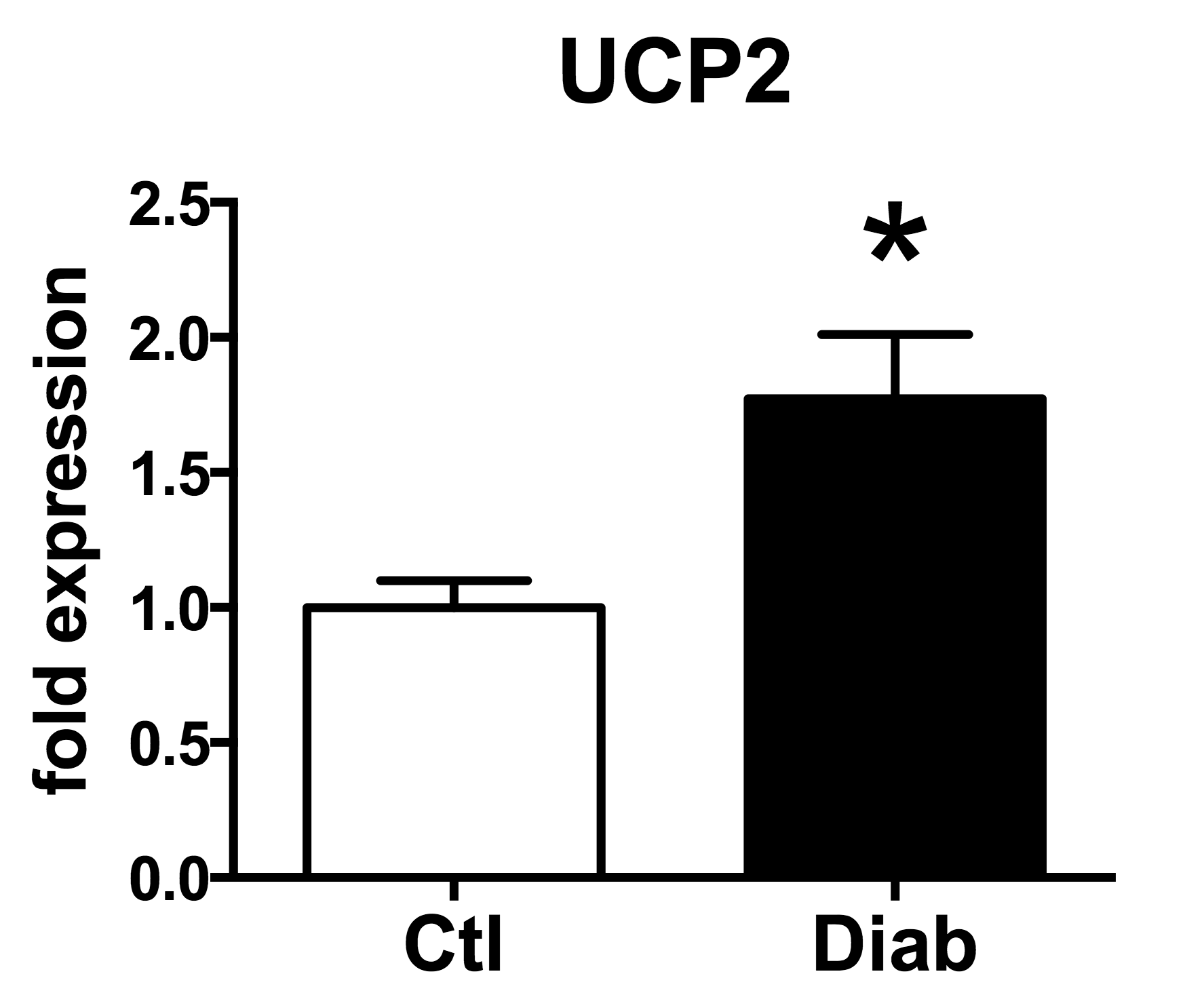

Supplement: S4 Fig — Diabetes was induced in 8-week old NMRI male mice (Charles River) by multiple low-dose streptozotocin (50 mg/kg on 5 consecutive days) protocol. Hyperglycemia was confirmed 2 weeks following the streptozotocin injections and muscle samples were collected. Gene expression was measured by UCP2 (assay ID: Mm00627598, Life Technologies) Taqman assay in combination with the normalization signal (VIC-labeled 18S rRNA control reagents, Cat# 438329, Life Technologies). Normalized expression values are shown. (*p<0.05 Diabetes induced significant increase in UCP2 expression, n = 6/group.) (TIFF) [file pone.0154813.s004.tiff]
