# Supplementary material for: Glucocorticoids Suppress Mitochondrial Oxidant Production via Upregulation of Uncoupling Protein 2 in Hyperglycemic Endothelial Cells
Source: PLoS One. 2016 Apr 29;11(4):e0154813. doi: 10.1371/journal.pone.0154813 (PMC4851329; doi:10.1371/journal.pone.0154813)
Supplement: S1 File — (DOC) [file pone.0154813.s005.doc]

# Supplementary methods

## Gene expression measurements in b.End3 cells exposed to hyperglycemia and dexamethasone

The expression of the nuclear encoded glucocorticoid receptor (GR), sirtuin 1 (SIRT) and cytochrome C (Cyt C) and the two mitochondrial encoded genes: the cytochrome c oxidase III (COX3) and 16S ribosomal RNA (16S RNA) was measured by real-time PCR. b.End3 cells were exposed to hyperglycemia or maintained at normoglycemia for 7 days and treated with dexamethasone (3 μM) for 3 days. RNA was isolated and reverse transcribed as described under “siRNA mediated gene silencing and real-time PCR measurements”. The primers in the below table were used in PCR reactions utilizing the SYBR Green method at 0.4 μM on a CFX96 thermocycler (Bio-Rad, Hercules, CA). Expression values were normalized to the amount of 18S rRNA (Life Technologies, Carlsbad, CA).

| **Target Gene** | **Primer**  **Orientation** | **Sequence** |
| --- | --- | --- |
| 16S RNA | forw. primer | 5’-AAACAGCTTTTAACCATTGTAGGC-3’ |
| 16S RNA | rev. primer | 5’-TTGAGCTTGAA GCTTTCTTTA-3’ |
| COX3 | forw. primer | 5’-AGACGTAATTCGTGAAGGAACC-3’ |
| COX3 | rev. primer | 5’-CCGAGACGATGAATAGAATTATACC-3’ |
| Cyt C | forw. primer | 5’-AAATCTCCACGGTCTGTTCG-3’ |
| Cyt C | rev. primer | 5’-CCAGGTGATGCCTTTGTTCT-3’ |
| GR | forw. primer | 5’-TTACCCCTACCCTGGTGTCA-3’ |
| GR | rev. primer | 5’-AAGGGTCATTTGGTCATCCA-3’ |
| SIRT | forw. primer | 5’-AAAAGATAATAGTTCTGACTGGAGCTG-3’ |
| SIRT | rev. primer | 5’-GGCGAGCATAGATACCGTCT-3’ |

**Suppl. Table**. **The primers used for gene expression analysis in b.End3 cells.**

## DNA isolation and mitochondrial DNA content measurement

b.End3 cells were exposed to hyperglycemia or maintained at normoglycemic conditions for 7 days and subsequently treated with dexamethasone (1 μM) for 3 days. The cellular DNA was isolated by lysing the cells in lysis buffer (100mM Tris pH 7.6, 20mM EDTA, 0.8% N-Lauroylsarcosine) supplemented with 25 U/ml RNase A and incubated at 37 °C for 1.5 hours. Subsequently, the samples were treated with Proteinase K (1 mg/ml) at 55 °C overnight and the DNA was extracted by phenol-chloroform extraction. The mitochondrial/genomic DNA content was determined by measuring the relative amount of the mitochondrial encoded Tyr tRNA coding region using the forward primer (0.1 μM): 5’-CACCTTAAGACCTCTGGTAAAAAGA-3’, reverse primer (0.1 μM): 5’-TGAGAATAATCAACGATTAATGAACA-3’ and probe (0.2 μM): 5’-FAM-CAGTCTAATGCTTACTCAGCCATTTT-3’ Iowa Black FQ, and the 129SV myosin heavy chain (*MYH*) promoter region (genomic DNA) by forward primer (0.4 μM): 5’-CAAGTAGGTCCGCAGCTAGG-3’, reverse primer (0.4 μM): 5’-GCACCCCAGCTTCACTTTTA-3’ and probe (0.4 μM) : 5’ TEX615-CTGCTGTGTTTGCAGAACAGCCT-3’ Iowa Black RQ in 20 ng input DNA in a dual Taqman assay.
